# New Isocoumarins from the Marine Fungus *Phaeosphaeriopsis* sp. WP-26

**DOI:** 10.3390/md21030150

**Published:** 2023-02-25

**Authors:** Pei Wang, Huifang Wang, Juchun Yang, Li Yang, Caihong Cai, Jingzhe Yuan, Fei Wu, Cuijuan Gai, Wenli Mei, Haofu Dai

**Affiliations:** 1 Key Laboratory of Research and Development of Natural Product from Li Folk Medicine of Hainan Province, Hainan Institute for Tropical Agricultural Resources, Institute of Tropical Bioscience and Biotechnology, Chinese Academy of Tropical Agricultural Sciences, Haikou 571101, China; 2 School of Chemistry and Chemical Engineering, Guangxi Minzu University, Nanning 530000, China

**Keywords:** isocoumarins, marine fungus, *Phaeosphaeriopsis* sp., neuroprotective effect, cytotoxicity

## Abstract

Five new isocoumarins, phaeosphaerins A–E (**1**–**5**), were isolated from the fermentation broth of the marine fungus *Phaeosphaeriopsis* sp. WP-26, along with one known isocoumarin, 6,8-dihydroxy-7-methoxy-3-methylisocoumarin (**6**), and two known pimarane-type diterpenes, diaportheins A (**7**) and B (**8**). Their structures were elucidated via NMR experiments, X-ray diffraction analysis, and comparison of the experimental and computed ECD curves. Compounds **1**–**7** displayed weak neuroprotective effects against H_2_O_2_-induced damage in SH-SY5Y cells. Moreover, compound **8** showed cytotoxicity against BEL-7402, SGC-7901, K562, A549, and HL-60 cell lines.

## 1. Introduction

Natural products are still currently considered as the best options for finding novel agents/active templates [1]. As an important source of natural products, marine fungi have provided a great deal of new active compounds [2,3,4]. *Phaeosphaeriopsis* sp. was first described as a new genus of fungi in 2003 [5], being obtained from marine and plant resources [6,7]. This genus of fungi has mainly been investigated for its plant-protective effects [8,9] and microbial taxonomy [5,10,11]. However, their metabolites are rarely studied, and up to now there has only been one paper about the metabolites of *Phaeosphaeriopsis* sp. [7]. 

**Figure 1:**
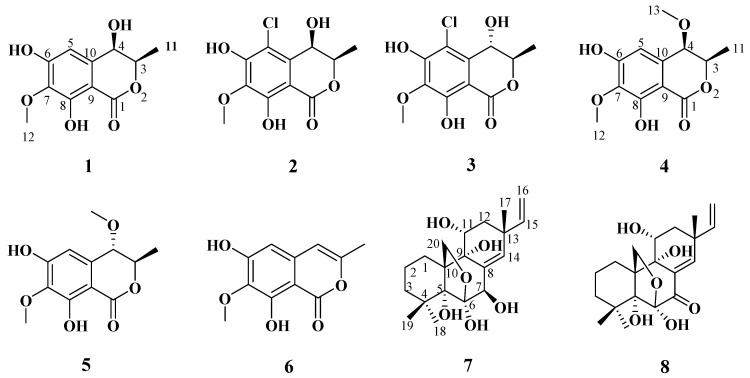
Chemical structures of compounds **1**–**8**.

In our ongoing investigation into the chemical diversity of marine fungi for marine drug discovery, a marine-derived fungus identified as *Phaeosphaeriopsis* sp. WP-26 was isolated from *Strombus luhuanus* Linnaeus. We studied the active metabolites of the marine-derived fungus *Phaeosphaeriopsis* sp. WP-26. During initial chemical screening of this fungus via HPLC-UV analysis, the ethyl acetate extract of fermentation broth of this strain exhibited a series of peaks showing attractive structure diversity, with UV absorptions different to those of the compounds reported in reference [7]. A detailed chemical investigation of this strain led to the isolation of five new isocoumarins (**1**–**5**) (Figure 1), along with one known isocoumarin, 6,8-dihydroxy-7-methoxy-3- methylisocoumarin (**6**) [12], and two known pimarane-type diterpenes, diaportheins A (**7**) and B (**8**) [13,14]. Hence, their isolation, structure determination, and biological activities are reported.

## 2. Results

### 2.1. Structural Elucidation

**Figure 2:**
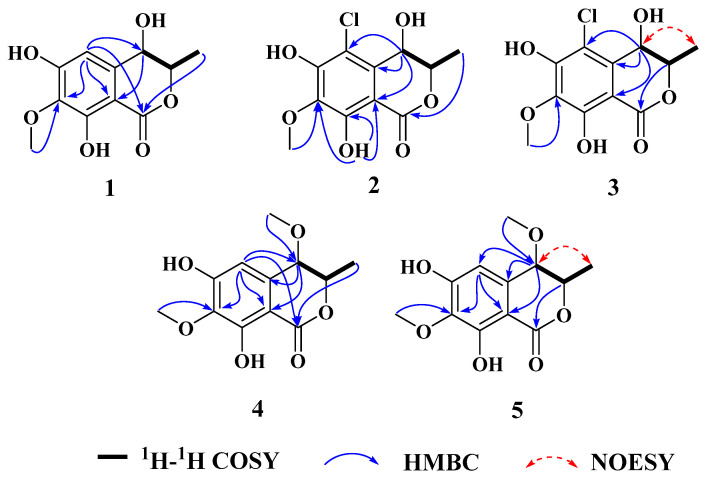
The key ^1^H-^1^H COSY, HMBC, and 1D NOESY correlations of chemical structures of compounds **1**–**5**.

**Figure 3:**
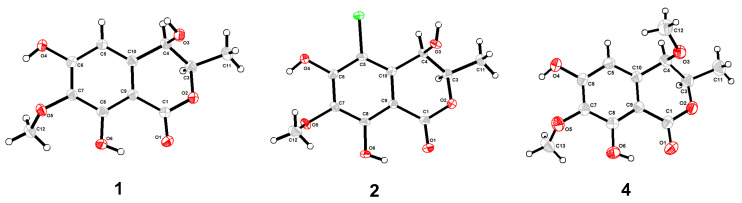
Molecular plots of compounds **1**, **2**, and **4**.

**Figure 4:**
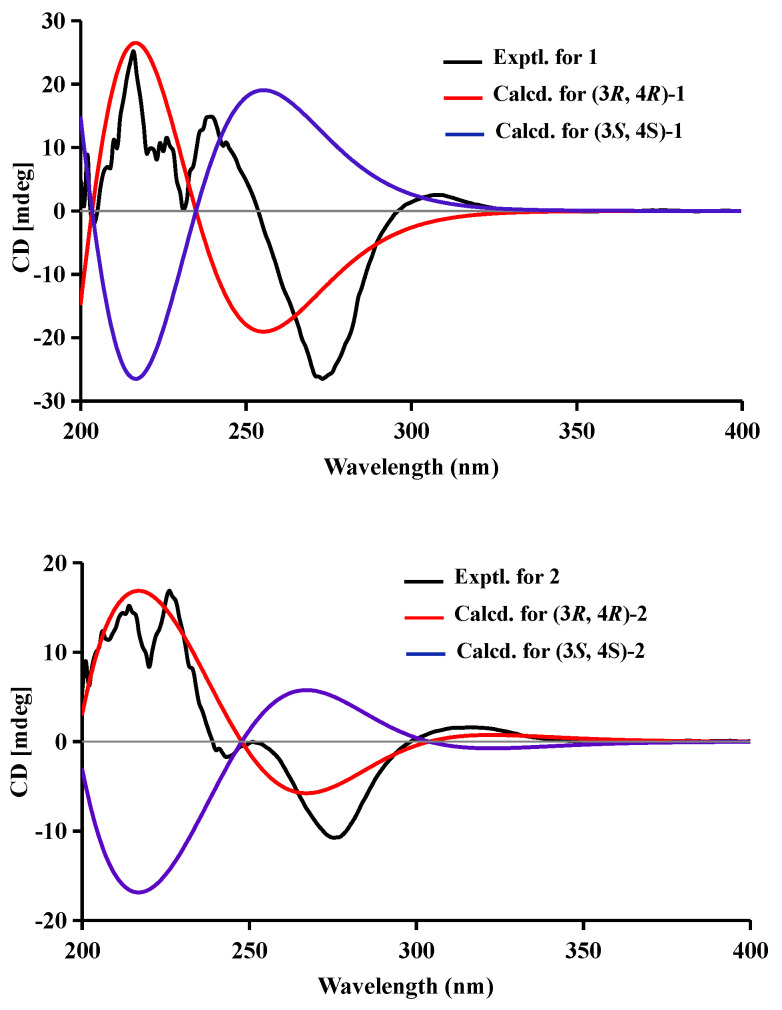

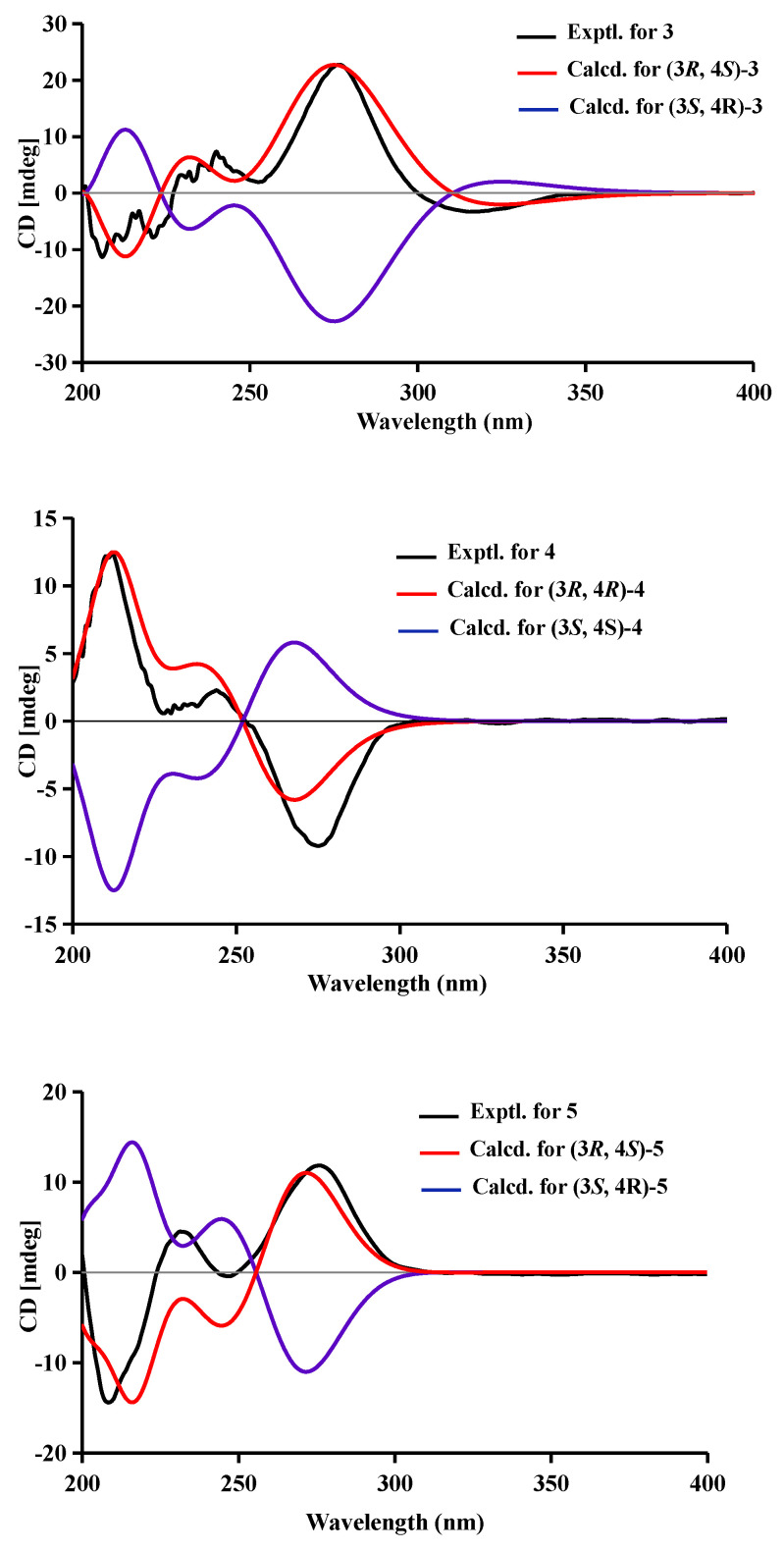
Experimental and calculated ECD spectra of compounds **1**–**5**.

Compound **1** was obtained as a brown powder with the molecular formula of C_11_H_12_O_6_ based on HRESIMS (*m*/*z* 263.0537 [M + Na]^+^, calcd. for C_11_H_12_O_6_Na^+^: 263.0526). Analysis of the ^1^H, ^13^C NMR and HSQC data revealed two oxygenated and one aromatic methines (*δ*_C/H_ 79.8/4.63, *δ*_C/H_ 67.6/4.42, and *δ*_C/H_ 108.5/6.49), one methyl (*δ*_C/H_ 16.3/1.48), one methoxy (*δ*_C/H_ 60.9/3.84), five aromatic quaternary carbons (*δ*_C_ 158.4, *δ*_C_ 157.4, *δ*_C_ 139.1, *δ*_C_ 136.2, and *δ*_C_ 101.3), and an ester carbonyl group (*δ*_C_ 171.3). These data were found to be very similar to those of the previously reported lignicol [15] through a detailed comparison between them. The COSY correlations (Figure 2) from H-4 to H_3_-11 through H-3, together with the HMBC correlations (Figure 2) from H-5 to C-1 (*δ*_C_ 171.3), C-4 (*δ*_C_ 67.6), C-7 (*δ*_C_ 136.2), and C-9 (*δ*_C_101.3), from H-4 to C-9 and C-10 (*δ*_C_ 139.1), from H_3_-12 to C-7 (*δ*_C_ 136.2), and from H_3_-11 to C-1 (weak correlation), suggested **1** had the same planar chemical structure as lignicol, while their different coupling constants between H-3 and H-4 (1.8 Hz) in **1** and lignicol (8 Hz), as well as the significant chemical shift differences in C-3 (1.6), C-4 (1.9), C-5 (1.5), C-10 (2.1), and C-11 (1.8), indicated they were a pair of epimers. To support the above structure elucidation and determine the absolute configuration of **1**, a single-crystal X-ray diffraction pattern was obtained using the anomalous scattering of Cu Kα radiation (Figure 3), allowing an explicit assignment of the absolute configuration of **1** as 3*R* and 4*R*. Additionally, the well-matched calculated and experimental ECD spectra of **1** also confirmed its absolute configuration (Figure 4). So, the structure of compound **1** was identified as that in Figure 1, and it was named phaeosphaerin A.

Compound **2**, a brown powder, gave a [M + Na]^+^ peak at *m*/*z* 297.1035 and a [M + 2 + Na]^+^ peak at *m*/*z* 299.0108 in the ratio of 3:1 in HRESIMS, indicating the presence of one chlorine atom in **2**. So, the molecular formula of **2** was determined as C_11_H_11_ClO_6_. The ^1^H NMR, ^13^C NMR, and HSQC spectra of **2** revealed resonances for a methyl (*δ*_C/H_ 16.5/1.53), a methoxy (*δ*_C/H_ 61.1/3.87), two oxygenated methines (*δ*_C/H_ 79.6/4.62 and *δ*_C/H_ 64.7/4.81), six aromatic quaternary carbons (*δ*_C_ 156.0, *δ*_C_ 154.9, *δ*_C_ 137.0, *δ*_C_ 135.6, *δ*_C_ 112.5, and *δ*_C_ 101.9), and an ester carbonyl carbon (*δ*_C_ 171.0), a phenolic hydroxyl chelated to the lactone carbonyl (*δ*_H_ 11.4). A detailed comparison of the aforesaid data and those of compound **1** suggested they had similar NMR data, except for the fact that an sp^2^ quaternary carbon (*δ*_C_ 112.5) in **2** replaced an sp^2^ methine (*δ*_C/H_ 108.5/6.49) in **1**. The above data combined with the COSY correlations (Figure 2) from H-4 to H_3_-11 through H-3, together with HMBC correlations (Figure 2) from H-4 to C-5 (*δ*_C_ 112.5), C-9 (*δ*_C_ 101.9), C-10 (*δ*_C_ 135.6), from H_3_-11 to C-1 (*δ*_C_ 171.0), H_3_-12 to C-7 (*δ*_C_ 137.0), and 8-OH to C-7, C-8 (*δ*_C_ 156.0) and C-9, suggested **2** had a similar structure to **1** and their only difference was that an aromatic proton in **1** was substituted by a Cl atom in **2**. The above assignment was further confirmed by a single-crystal X-ray diffraction pattern obtained using the anomalous scattering of Mo Kα radiation (Figure 3). The absolute configuration of **2** was determined as 3*R* and 4*R* according to the single-crystal X-ray crystallographic analysis, which was consistent with the result of ECD calculation (Figure 4). Hence, the structure of compound **2** was identified as that shown in Figure 1 and it was named as phaeosphaerin B.

Compound **3** was obtained as a brown oil. It had the same molecular formula as **2,** which was determined as C_11_H_11_ClO_6_, based on the characteristic protonated molecular ions at *m*/*z* 297.0147 [M + Na]^+^ (calcd. for C_11_H_11_ClO_6_Na^+^: 297.0136) and 299.0121 [M + Na + 2]^+^ in the ratio of 3:1 in the HRESIMS spectrum. A comparison of the NMR data of **3** (Table 1 and Figure 2) and **2** suggested they had the same planar structure but significant chemical shift differences in C-1(1.8), C-3 (2.6), C-4 (1.3), C-5 (1.2), C-10 (2.5), and C-11 (1.4) as well as the 1D NOE correlation from H-4 (*δ*_H_ 4.91) to H_3_-11 (*δ*_H_ 1.25) (Figure S19 in Supplementary Information), indicating that they were a pair of epimers. So, the relative configuration of **3** was determined as 3R* and 4S*. The absolute configuration of **3** was identified via a comparison of experimental and calculated ECD spectra (Figure 4), and the well-matched calculated and experimental ECD spectra of **3** suggested the 3*R*, 4*S* configurations of **3**. Thus, the structure of compound **3** was identified as that shown in Figure 1, and it was named phaeosphaerin C. 

Compound **4** was also obtained as a brown powder. Its molecular formula was identified as C_12_H_14_O_6_ according to HRESIMS with a peak at *m*/*z* 277.0692 [M + Na]^+^ (calcd. for C_12_H_14_NaO_6_^+^:277.0683). The NMR data of **4** (Table 2) were very similar to those of **1** except for the presence of an extra methoxy group (δ_C/H_ 56.8/3.28) in **4**. The aforementioned data indicated that the difference between the planar structure of **4** and that of **1** was a hydroxyl group in **1** substituted by a methoxy in **4**, as evidenced by the COSY correlations (Figure 2) from H-4 to H_3_-11 through H-3, together with HMBC correlations from H-5 to C-1 (*δ*_C_ 171.1), C-4 (*δ*_C_ 76.3), C-7 (*δ*_C_ 136.6), and C-9 (*δ*_C_ 101.4), from H-4 to C-9 and C-10 (*δ*_C_ 135.6), from H_3_-12 to C-7, from H_3_-11 to C-1 (weak correlation), and from H_3_-13 to C-4. The above assignment was further confirmed via a single-crystal X-ray diffraction pattern obtained using the anomalous scattering of Cu Kα radiation (Figure 3), which also led to an unambiguous assignment of the absolute configuration of **4** as 3*R* and 4*R.* The absolute configuration of **4** was also verified by its well-matched calculated and experimental ECD spectra (Figure 4). So, the structure of compound **4** was identified as that shown in Figure 1 and it was named phaeosphaerin D. 

Compound **5** was obtained as a brown oil. Its positive-ion HRESIMS data, revealing protonated molecular ions at *m*/*z* 255.0863 [M + H]^+^ and *m*/*z* 277.0688 [M + Na]^+^, corresponded to a molecular formula of C_12_H_14_O_6_ (calcd. for C_12_H_15_O_6_^+^:255.0863 and C_12_H_14_NaO_6_^+^:277.0683), which was the same as that of **4**. A detailed comparison of the 1D and 2D NMR data (Table 2 and Figure 2) of **5** and **4** suggested that they have the same planar structure, while the significant chemical shift differences in C-1 (1.4), C-4 (1.9), C-10 (1.2), and C-11 (1.4) between them and the 1D NOE correlation from H-4 (*δ*_H_ 4.11) to H_3_-11 (*δ*_H_ 1.30) indicated that they were also a pair of epimers, just like **2** and **3**. So, the relative configuration of **5** was determined as 3R* and 4S*. Then, the calculated ECD curve for **5** was found to match well with the experimental one (Figure 4), and so we assigned the absolute configurations of **5** as 3*R* and 4*S.* Thus, the structure of compound **5** was identified as that shown in Figure 1, and it was named phaeosphaerin E.

Besides the above new compounds **1**–**5**, three known compounds were also isolated, and indentified as isocoumarins 6,8-dihydroxy-7-methoxy-3-methylisocoumarin (**6**) [12] and two known pimarane-type diterpenes, diaportheins A (**7**) and B (**8**) [13,14] by comparing their NMR spectroscopic (Tables S1–S3 in Supplementary Information) and physical data with literature values. 

### 2.2. The Bioactivities of Compounds ***1***–***8*** from Phaeosphaeriopsis sp. WP-26

**Figure 5:**
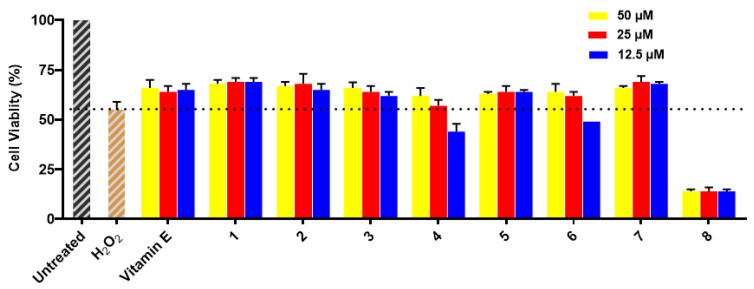
Neuroprotective effects of **1**–**8** on H_2_O_2_-induced (1000 μM) SH-SY5Y cell injury. The percentage of surviving cells is presented as the percentage of viable cells compared to the untreated group (cell viability 100%). All data are presented as the means of three independent experiments. Vitamin E was used as a positive control (cell viability 66%, 64%, and 65%, respectively).

Compounds **1**–**8** were evaluated for their neuroprotective effects against H_2_O_2_-induced damage in SH-SY5Y cells. The results exhibit that cell viability was remarkably reduced to around 55% in H_2_O_2_-treated cells compared to the cells of the untreated group, in which cell viability was 100%. Compounds **1**–**3**, **5**, and **7** showed similar protective activities for SH-SY5Y cells with H_2_O_2_-induced injury to those of the positive control vitamin E at all test concentrations (see Figure 5), while compounds **4** and **6** showed only neuroprotective activities at 50 μM and 25 μM. Compound **8** did not show neuroprotective activity at any test concentration because of its cytotoxicity.

In addition, compounds **1**–**8** were evaluated for their cytotoxicity against BEL-7402, SGC-7901, K562, A549, and HL-60 cell lines; however, only the previously reported compound diaporthein B (**8**) exhibited cytotoxicity against BEL-7402, SGC-7901, K562, A549, and HL-60 cell lines, with IC_50_ values of 38, 19, 8.5, 12, and 16 μM, respectively. 

## 3. Materials and Methods 

### 3.1. General Experimental Procedures

Optical rotations were recorded with a Modular Circular Polarimeter 500 polarimeter (Anton Paar, Austria). ECD and UV spectra were measured on an MOS-500 spectrometer (Biologic, France). NMR spectra were recorded on a Bruker AV III spectrometer (Bruker, Billerica, MA, USA) (^1^H NMR at 500 MHz and ^13^C NMR at 125 MHz for **1**–**3**, **6**, **7**, and **8**, ^1^H NMR at 600 MHz and ^13^C NMR at 150 MHz for compounds **4** and **5**) using TMS as the internal standard. HRESIMS spectra were measured on an ESI-Q-TOF Pulsar mass spectrometer (Bruker, Germany). Semipreparative HPLC was performed using an ODS column (COSMOSIL-packed, 5 μm, 250 mm × 10 mm, 4 mL/min). TLC and column chromatography (CC) were performed on plates pre-coated with silica gel GF254 (10−40 μm) and over silica gel (200−300 mesh, Qingdao Marine Chemical Factory). Sephadex LH-20 (Merck, Germany) and ODS gel (20–45 µm, Fuji Silysia Chemical Co. Ltd., Aichi-ken, Japan) were used for column chromatography.

### 3.2. Collection and Phylogenetic Analysis

The fungus strain, *Phaeosphaeriopsis* sp. WP-26, was isolated from a *Strombus luhuanus* Linnaeus collected from Yagong Island of the Xisha Islands in the South China Sea in August 2020. The sample (1.0 g) was diluted to 10^−2^ g/mL with sterile H_2_O after grinding, and then a 100 µL supernate was deposited on a Potato Dextrose Agar (PDA) (200.0 g of potato, 20.0 g of glucose, 20.0 g of agar per liter, 33.0g of NaCl, 1.0 L of water) plate containing chloramphenicol (100 µg/mL) as a bacterial inhibitor. A reference culture was maintained in our laboratory at −80 °C. Working stocks were prepared on PDA slants stored at 4 °C.

The fungus was identified based on the DNA sequences, which were deposited in the Genome Sequence Archive (Genomics, Proteomics & Bioinformatics 2021) in the National Genomics Data Center (Nucleic Acids Res 2022), the China National Center for Bioinformation / Beijing Institute of Genomics, Chinese Academy of Sciences (GSA: CRA009121. The ITS gene sequence data are provided in the Supplementary Materials), and are publicly accessible at https://ngdc.cncb.ac.cn/gsa (accessed on 2 December 2022). The mycelium was ground to a fine powder in liquid N_2_; then, genomic DNA was extracted, and the TIS region was amplified via PCR using primers ITS1 (GTAG TCATATGCTTGTCTC) and ITS4 (GCATCACAG ACCTG TTATTGCCTC). PCR products were sequenced on an Applied Biosystems 3730 XL Genetic Analyzer (Applied Biosystems Inc., Foster City, CA, USA).

### 3.3. Cultivation and Extraction

The spores of *Phaeosphaeriopsis* sp. WP-26 were directly transferred to 150 mL of a liquid medium (the potato liquid media consisting of 200.0 g/L potato, 20.0 g/L glucose, and 1000 mL deionized water) in Erlenmeyer flasks (500 mL) and shaken for 48 h (28 ± 0.5 °C, 180 rpm). Further, 5 mL of seed broth was transferred aseptically to 1000 mL Erlenmeyer flasks (160 flasks), each containing rice medium (80 g of rice and 120 mL of water). The flasks were incubated at room temperature under static conditions for 30 days. Then, the cultures were extracted three times via EtOAc and concentrated in vacuo to obtain a 138.5 g EtOAc extract.

### 3.4. Purification

The EtOAc extract (138.5 g) was suspended in 90% MeOH aqueous solution, and then the suspension was extracted three times using petroleum ether. The remaining MeOH aqueous part was concentrated in vacuo to obtain a 70.5 g extract.

The crude extract (70.5 g) was separated into 18 fractions (Fr.1−Fr.18) on a silica gel VLC column using step gradient elution with CH_2_Cl_2_−MeOH (100%−0). Compound **8** (15.0 mg) was obtained via recrystallization from Fr.3 in MeOH. Fr.4 was separated into eight fractions (Fr.4.1−Fr.4.8) via a reversed-phase silica gel column and eluted with a step gradient of MeOH−H_2_O (20−100%). Fr.4.3 was further separated into three fractions (Fr.4.3.1−Fr.4.3.3) via Sephadex LH-20 eluted with MeOH. Fr.4.3.2 was purified by semipreparative HPLC on an ODS column using the solvent system of 35% MeOH aqueous solution (65% water added to 0.05% trifluoracetic acid) to yield compound **1** (8 mg, *t*_R_ 18 min), compound **2** (8.0 mg, *t*_R_ 30 min), and compound **3** (3 mg, *t*_R_ 28 min). Fr.4.4 was purified via semipreparative HPLC on an ODS column using the solvent system of 28% MeCN aqueous solution (72% water added to 0.05% trifluoracetic acid) to yield compound **5** (3 mg, *t*_R_ 2.2 min). Fr.4.5 (5.10 g) was fractionated into 18 subfractions (Fr.4.1−Fr.4.18) on a reversed-phase silica gel column and eluted with a step gradient of MeOH−H_2_O (25−100%). Fr.4.5.7 was further separated via semipreparative HPLC on an ODS column using the solvent system of 40% MeCN aqueous solution (60% water added to 0.05% trifluoracetic acid) to yield compound **4** (2.5 mg, *t*_R_ 26.2 min). Fr.4.5.9 (98 mg) was purified via semipreparative HPLC on an ODS column and eluted with a solvent system of 25% MeCN aqueous solution (75% water added to 0.05% trifluoracetic acid) to yield compound **6** (4.0 mg, *t*_R_ 28.0 min). Fr.4.5.13 was purified via semipreparative HPLC on an ODS column using the solvent system of 50% MeOH aqueous solution (50% water added to 0.05% trifluoracetic acid) to yield compound **7** (6.0 mg, *t*_R_ 19.6 min).

### 3.5. Characterization of the Compounds

Phaeosphaerin A (**1**): brown powder; [α]_D_^25^ −15.0 (*c* 0.10, MeOH); ECD (1.4 mM, MeOH) λ_max_ (Δε) 216 (+5.6), 239 (+3.27), 273 (−5.84), 309 (+0.55) nm; UV (MeOH) λ_max_ (log ε) 234 (2.80), 276 (2.91) nm; IR (KBr)ν_max_ 3421, 1638, 1589, 1502 cm^−1^; ^1^H and ^13^C NMR, see Table S1; HRESIMS *m/z* 263.0537 [M +Na]^+^ (calcd. for C_11_H_12_NaO_6_, 263.0526).

Phaeosphaerin B (**2**): brown powder; [α]_D_^20^ −6.0 (*c* 0.10, MeOH); ECD (0.5 mM, MeOH) λ_max_ (Δε) 213 (+9.38), 226 (+10.1), 244 (−0.86), 276 (−7.0), 316 (+1.05) nm; UV (MeOH) λ_max_ (log ε) 235 (3.39), 275 (3.17), 321 (2.87) nm; IR (KBr)ν_max_ 3430, 1671, 1620, 1573, 1462 cm^−1^; ^1^H and ^13^C NMR, see Table 1; HRESIMS *m/z* 297.0135 [M + Na]^+^ (calcd. for C_11_H_11_ClNaO_6_, 297.0136).

Phaeosphaerin C (**3**): brown oil; [α]_D_^25^ +35.0 (*c* 0.1, MeOH); ECD (1.0 mM, MeOH) λ_max_ (Δε) 211 (−2.95), 224 (−3.1), 246 (+1.1), 276 (+4.7), 316 (−0.67) nm; UV (MeOH) λ_max_ (log ε) 232 (3.32), 278 (2.96), 323 (2.71) nm; IR (KBr)ν_max_ 3440, 1670, 1456 cm^−1^; ^1^H and ^13^C NMR, see Table 1; HRESIMS *m/z* 297.0147 [M + Na]^+^ (calcd. for C_11_H_11_ClNaO_6_, 297.0136).

Phaeosphaerin D (**4**): brown powder; [α]_D_^20^ −14.0 (*c* 0.10, MeOH); ECD (1.2 mM, MeOH) λmax (Δε) 211 (+4.63), 243 (+0.93), 276 (−4.42) nm; UV (MeOH) λmax (log ε) 231 (3.06), 276 (2.92), 308 (2.5) nm; IR (KBr)ν_max_ 3357, 1652, 1594, 1510 cm^−1^; ^1^H and ^13^C NMR, see Table 1; HRESIMS *m*/*z* 277.0692 [M + Na]^+^ (calcd. for C_12_H_14_NaO_6_, 277.0683).

Phaeosphaerin E (**5**): brown oil; [α]_D_^20^ +18.6 (*c* 0.07, MeOH); ECD (1.0 mM, MeOH) λmax (Δε) 210 (−2.00), 233 (+0.67), 276 (+1.82) nm; UV (MeOH) λmax (log ε) 232 (2.8), 276 (2.6), 312 (2.5) nm; IR (KBr)ν_max_ 3402, 1678, 1518 cm^−1^; ^1^H and ^13^C NMR, see Table 1; HRESIMS *m*/*z* 255.0863 [M + H]^+^, *m*/*z* 277.0688 [M + Na]^+^ (calcd. for C_12_H_15_O_6_, 255.0863; C_12_H_14_O_6_Na, 277.0683).

### 3.6. X-ray Crystallographic Analysis

Compound **1** was obtained as a colorless crystal with the molecular formula of C_11_H_12_NO_6_ from MeOH. Space group P21 (no. 4), *a* = 6.5197(2) Å, *b* = 4.51930(10) Å, *c* = 17.8784(5) Å, *α* = 90.00°, *β* = 96.176(2)°, *γ =* 90.00°, *V* = 523.72(2) Å^3^, *Z* = 2, *D*_calc_ = 1.523 g/cm^3^, *T* = 169.99(10) K, *μ*(Cu Kα) = 1.077 mm^−1^, F(000) *=* 252.0, crystal size 0.16 × 0.13 × 0.12 mm, *R*_1_ = 0.0284 (I > 2σ(I)), *wR*_2_ = 0.0785 (all data), Flack parameter = 0.06(7). 3219 reflections measured (4.972° ≤ 2Θ ≤ 147.062°), 2003 unique (Rint = 0.0166, Rsigma = 0.0163) which were used in all calculations. The structure was solved with direct methods (SHELXS-97) and expanded using Fourier techniques (SHELXL-97). Crystallographic data (excluding structure factors) for structure **1** in this paper have been deposited in the Cambridge Crystallographic Data Center under supplementary publication number CCDC 2220024.

Compound **2** was obtained as a colorless crystal with the molecular formula of C_11_H_11_ClO_6_ from MeOH. Space group P2_1_2_1_2_1_, *a* = 8.2724(4) Å, *b* = 8.8125(4) Å, c = 15.7345(6) Å, α = 90.00°, β = 94.117(2)°, *γ* = 90.00°, *V* = 1147.05(9) Å^3^, Z = 4, *D*_calc_ = 1.590 g/cm^3^, *T* = 149.99(10) K, *μ*(Mo Kα) = 0.351 mm^−1^, F(000) *=* 504.0, crystal size 0.15 × 0.13 × 0.12 mm, *R*_1_ = 0.0334 (I > 2σ(I)), *wR*_2_ = 0.0735 (all data), 8945 reflections measured (5.178° ≤ 2Θ ≤ 59.124°), 2740 unique (Rint = 0.0340, Rsigma = 0.0376), which were used in all calculations. The structure was solved with direct methods (SHELXS-97) and expanded using Fourier techniques (SHELXL-97). Crystallographic data (excluding structure factors) for structure **2** in this paper have been deposited in the Cambridge Crystallographic Data Center under supplementary publication number CCDC 2220048.

Compound **4** was obtained as a colorless crystal with the molecular formula of C_12_H_14_O_6_ from MeOH. Space group R3, *a* = 37.3267(10) Å, *b* = 37.3267(10)Å, *c* = 4.66340(10) Å, α = 90.00°, β = 90.0°, *γ* = 120.00°, *V* = 5626.9(3) Å^3^, Z = 18, *D*_calc_ = 1.350 g/cm^3^, *T* = 169.99(10) K, *μ*(Cu Kα) = 0.932 mm^−1^, F(000) *=* 2412.0, crystal size 0.14 × 0.11 × 0.09 mm, R1 = 0.0428 (I > 2σ(I)), wR2 = 0.0989 (all data), Flack parameter = -0.18(12). 25461 reflections measured (4.734°≤ 2Θ ≤ 149.448°),4915 unique (Rint = 0.0413, Rsigma = 0.0271), which were used in all calculations. The structure was solved with direct methods (SHELXS-97) and expanded using Fourier techniques (SHELXL-97). Crystallographic data (excluding structure factors) for structure **4** in this paper have been deposited in the Cambridge Crystallographic Data Center under supplementary publication number CCDC 2220209.

### 3.7. ECD Calculation

The preliminary conformational search for **1**–**5** was carried out in Confab [16] using an MMFF94 molecular mechanics force field. The Gaussian 16 package was used to carry out the calculations using the density functional theory (DFT) [17]. The obtained conformers were optimized at the B3LYP/6-31G (d) level, and frequency analysis was also performed at the same level. More accurate energies of optimized conformers were evaluated at the M06-2X/Def2-TZVP level in the methanol, and were then added to the thermal correction of Gibbs free energies obtained with frequency analyses to afford the Gibbs free energies of each conformer. Then, the ECD spectrum was calculated for the optimized conformers using the TDDFT method at the APFD/6-311+G (2d) level with the IEFPCM model in MeOH. The ECD spectra were simulated using the versatile web server provided by Yinfo Information Technology Co., Ltd. (https://cloud.yinfotek.com, accessed on 1 February 2023) with a half-bandwidth of 0.29–0.50 eV and a UV shift (−17, −1, 9, 0, 3) for **1**–**5**. Finally, the calculated ECD spectra were compared with the experimental data. 

### 3.8. Neuroprotective Properties against H_2_O_2_-Induced Damage In Vitro

The neuroprotective properties of compounds **1**–**8** against H_2_O_2_-induced damage in SH-SY5Y cells were assayed using the MTT method [18]. The SH-SY5Y cells were cultured in 96-well plates for 24 h. Next, the cells were treated with the tested compounds at the test concentrations (50, 25, and 12.5 μM) for 12 h, and then exposed to 1000 μM H_2_O_2_ for 12 h. After adding 20 μL of MTT (5 mg/mL) to each well for 4 h, 150 μL of DMSO was added to dissolve the formazan crystals. Finally, absorbance was read at 490 nm with a Synergy H1 microplate reader (BioTek, Winooski, VT, USA). The cell viability was expressed as a percentage with the control group as 100%.

### 3.9. Cytotoxicity Assay

The MTT method optimized by Mosmann et al. [19] was performed in vitro to test the cytotoxic activity of compounds **1**–**8**. Adriamycin was used as a positive control with IC_50_ values of 3.0, 4.1, 4.2, 2.0, and 11 μM for the cell lines K562, BEL-7402, SGC-7901, A549, and Hela, respectively. Additionally, the medium without the test compound was used as a negative control in the bioassay.

## 4. Conclusions

In summary, six new isocoumarins (**1**–**5**), along with one known isocoumarin, 6,8- dihydroxy-7-methoxy-3-methylisocoumarin (**6**), and two known pimarane-type diterpenes, diaportheins A (**7**) and B (**8**), were isolated from the rice medium culture of the marine fungus *Phaeosphaeriopsis* sp. WP-26. Compounds **1**–**7** exhibited weak neuroprotective effects against H_2_O_2_-induced damage in SH-SY5Y cells. In addition, compound **8** showed cytotoxicity against the BEL-7402, SGC-7901, K562, A549, and HL-60 cell lines with IC_50_ values of 38 μM, 19 μM, 8.5 μM, 12 μM, and 16 μM, respectively. 

## Figures and Tables

**Table 1:** ^1^H (500 MHz) and ^13^C (125 MHz) NMR data for phaeosphaerins A–C (**1**–**3**) in CH_3_OH-*d*_4_.

Position	1		2		3	
	*δ*_C_, Type	*δ*_H_ Mult. (*J* in Hz)	*δ*_C_, Type	*δ*_H_ Mult. (*J* in Hz)	Δ_c_, Type	Δ_h_ Mult. (*J* in Hz)
1	171.3, C		171.0, C		169.2, C	
2						
3	79.8, CH	4.63, qd, (6.6, 1.8)	79.6, CH	4.62, qd, (6.6, 1.8)	82.2, CH	4.88, qd, (6.5, 1.9)
4	67.6, CH	4.42, d, (1.8)	64.7, CH	4.81, d, (1.8)	66.0, CH	4.91 ^a^, overlap
5	108.5, CH	6.49, s	112.5, CH		113.7, C	
6	158.4, C		154.9, C		155.1, C	
7	136.2, C		137.0, C		137.1, C	
8	157.4, C		156.0, C		156.1, C	
9	101.3, C		101.9, C		101.6, C	
10	139.1, C		135.6, C		133.1, C	
11	16.3, CH_3_	1.48, d, (6.6)	16.5, CH_3_	1.53, d, (6.6)	17.9, CH_3_	1.25, d, (6.5)
12	60.9, CH_3_	3.84, s	61.1, CH_3_	3.87, s	61.1, CH_3_	3.88, s
8-OH				11.4, s		

^a^ These data are from HMBC and HSQC spectra.

**Table 2:** ^1^H (600 MHz) and ^13^C (150 MHz) NMR data for phaeosphaerins D and E (**4** and **5**) in CH_3_OH-*d*_4_.

Position	4		5	
	*δ*_C_, Type	*δ*_H_ Mult. (*J* in Hz)	*δ*_C_, Type	*δ*_H_ Mult. (*J* in Hz)
1	171.1, C		169.7, C	
2				
3	79.4, CH	4.68, qd, (6.6, 1.9)	79.9, CH	4.83 ^a^, m
4	76.3, CH	4.05, d, (1.9)	78.2, CH	4.11, d, (3.0)
5	109.5, CH	6.50, (s)	109.9, CH	6.51, s
6	157.7, C		158.2, C	
7	136.6, C		136.6, C	
8	157.8, C		157.8, C	
9	101.4, C		101.3, C	
10	135.6, C		134.4, C	
11	16.4, CH_3_	1.50, d, (6.6)	17.8, CH_3_	1.30, d, (6.8)
12	60.9, CH_3_	3.85, (s)	60.9, CH_3_	3.86, s
13	56.8, CH_3_	3.28, (s)	57.2, CH_3_	3.36, s

^a^ These data are from HMBC and HSQC spectra.

## Data Availability

The original data presented in the study are included in the article and Supplementary Material.

## Supplementary Materials

The following are available online at https://www.mdpi.com/article/10.3390/md21030150/s1. Figures S1–S34: NMR spectra and HRESI spectra for compounds **1**–**5**. Tables S1–S3: 1D NMR data for compounds **6**–**8**.
