# Supplementary material for: New Isocoumarins from the Marine Fungus Phaeosphaeriopsis sp. WP-26
Source: Mar Drugs. 2023 Feb 25;21(3):150. doi: 10.3390/md21030150 (PMC10054857; doi:10.3390/md21030150)
Supplement: Supplementary file 1 [file marinedrugs-21-00150-s001.zip › marinedrugs-2146476-supplementary.pdf]

Supplementary Information

New Isocoumarins from the Marine Fungus *Phaeosphaeriopsis* sp. WP-26

Pei Wang [1,2], Huifang Wang [1], Juchun Yang [1], Li Yang [1], Caihong Cai [1], Jingzhe Yuan [1], Fei Wu [1], Cuijuan Gai [1], Wenli Mei [1,*] and Haofu Dai [1,*]

[1] Key Laboratory of Research and Development of Natural Product from Li Folk Medicine of Hainan Province, Hainan Institute for Tropical Agricultural Resources, Institute of Tropical Bioscience and Biotechnology, Chinese Academy of Tropical Agricultural Sciences, Haikou 571101, China

[2] School of Chemistry and Chemical Engineering, Guangxi Minzu University, Nanning 530000, China

* Correspondence: meiwenli@itbb.org.cn (W.M.); daihaofu@itbb.org.cn (H.D.); Tel./Fax: +86-0898-6698-7529 (W.M.); +86-0898-6696-1869 (H.D.)

Table of Contents

S1. The ITS gene sequences data of *Phaeosphaeriopsis* sp. WP-26……S4

Figure S1. ^{1}H NMR spectrum (500 MHz) for compound **1** in CH_3OH-d_4……S5

Figure S2. ^{13}C NMR and DEPT135 spectra (125 MHz) of compound **1** in CH_3OH-d_4……S5

Figure S3. HSQC spectrum (500 MHz) of compound **1** in CH_3OH-d_4……S6

Figure S4. ^{1}H-^{1}H COSY spectrum (500 MHz) of compound **1** inCH_3OH-d_4……S6

Figure S5. HMBC spectrum (500 MHz) of compound **1** in CH_3OH-d_4. ……S7

Figure S6. HRESIMS spectrum for compound **1**……S8

Figure S7. ^{1}H NMR spectrum (500 MHz) data for compound **2** in CH_3OH-d_4……S9

Figure S8. ^{13}C NMR and DEPT135 spectra (125 MHz) of compound **2** in DMSO-d_6……S9

Figure S9. HSQC spectrum (500 MHz) of compound **2** in CH_3OH-d_4……S10

Figure S10. ^{1}H-^{1}H COSY spectrum (500 MHz) of compound **2** in CH_3OH-d_4……S10

Figure S11. HMBC spectrum (500 MHz) of compound **2** in CH_3OH-d_4 ……S11

Figure S12. HRESIMS spectrum for compound **2**……S12

FigureS13. ^{1}H NMR spectrum (500 MHz) data for compound **3** in CH_3OH-d_4……S13

Figure S14. ^{13}C NMR and DEPT135 spectra (125 MHz) of compound **3** in DMSO-d_6……S13

Figure S15. HSQC spectrum (500 MHz) of compound **3** in CH_3OH-d_4……S14

Figure S16. ^{1}H-^{1}H COSY spectrum (500 MHz) of compound **3** in CH_3OH-d_4……S14

Figure S17. HMBC spectrum (500 MHz) of compound **3** in CH_3OH-d_4 ……S15

Figure S18. ROESY spectrum (500 MHz) of compound **3** in CH_3OH-d_4. ……S15

Figure S19. 1D NOESY spectrum (500 MHz) of compound **3** in CH_3OH-d_4. ……S16

Figure S20. HRESIMS spectrum for compound **3**……S17

Figure S21. ^{1}H NMR spectrum (600 MHz) data for compound **4** in CH_3OH-d_4……S18

Figure S22. ^{13}C NMR and DEPT 135 spectra (150 MHz) of compound **4** in CH_3OH-d_4……S18

Figure S23. HSQC spectrum (600 MHz) of compound **4** in CH_3OH-d_4……S19

Figure S24. ^{1}H-^{1}H COSY spectrum (600 MHz) of compound **4** in CH_3OH-d_4……S19

Figure S25. HMBC spectrum (600 MHz) of compound **4** in CH_3OH-d_4……S20

Figure S26. HRESIMS spectrum for compound **4**……S21

Figure S27. ^{1}H NMR spectrum (600 MHz) data for compound **5** in CH_3OH-d_4……S22

Figure S28. ^{13}C NMR and DEPT135 spectra (150 MHz) of compound **5** in DMSO-d_6……S22

Figure S29. HSQC spectrum (600 MHz) of compound **5** in CH_3OH-d_4……S23

Figure S30. ^{1}H-^{1}H COSY spectrum (600 MHz) of compound **5** in CH_3OH-*d_4*……………………..………S23

Figure S31. HMBC spectrum (600 MHz) of compound **5** in CH_3OH-*d_4*………………………………S24

Figure S32. ROESY spectrum (600 MHz) of compound **5** in CH_3OH-*d_4*. …………………………………S24

Figure S33. 1D NOESY spectrum (500 MHz) of compound **5** in CH_3OH-*d_4*. …………………………………S25

Figure S34. HRESIMS spectrum for compound **5**…………………………………………………………S26

Table S1. ^{1}H and ^{13}C NMR Data for compound **6** and 6,8-dihydroxy-7- methoxy-3-methylisocoumarin in CH_3OH-*d_4* ……………………………………………………………………………………………S27

Table S2.^{1}H and ^{13}C NMR Data for compound **7** and Diaporthein A in CHCl……………………………S27

Table S3. ^{1}H and ^{13}C NMR Data for compound **8**and Diaporthein B in $CHCl_3$-*d*…………………S28

S1. The ITS gene sequences data of *Phaeosphaeriopsis* sp. WP-26

CCTGCGGAAGGATCATTACATTCAGTAGCCCAGCTACTTGTTTACACCCTTGTCTTTTTGCGTACTTATC

GTTTCCTCGGCGGGCTTGCCTGCCGGTTGGACAACTTTATAACCTTTTTAAATCTTCAATCAGCGTCTG

AATAATATACAATAATTACAACTTTCAACAACGGATCTCTTGGTTCTGGCATCGATGAAGAACGCAGCG

AAATGCGATAAGTAGTGTGAATTGCAGAATTCAGTGAATCATCGAATCTTTGAACGCACATTGCGCCC

CTTGGTATTCCATGGGGCATGCCTGTTCGAGCGTCATTTGTACCTTCAAGCTTTGCTTGGTGTTGGGTG

CTTGTCTTTTTGTTAAGACTCACCTCAAAGTCATTGGCAGCCAGTGTTTTGGTAGTAAGCGCAGCACAT

TTTGCGTCTTGGTCCCTTAACAGCAGCATCCATCAAGCCATTTTCTCACTTTTGACCTCGGATCAGGTA

GGGATACCCGCTGAACTTAAGCATATCAAT

Figure S1. ^{1}H NMR spectrum (500 MHz) data for compound **1** in CH_3OH-*d*4

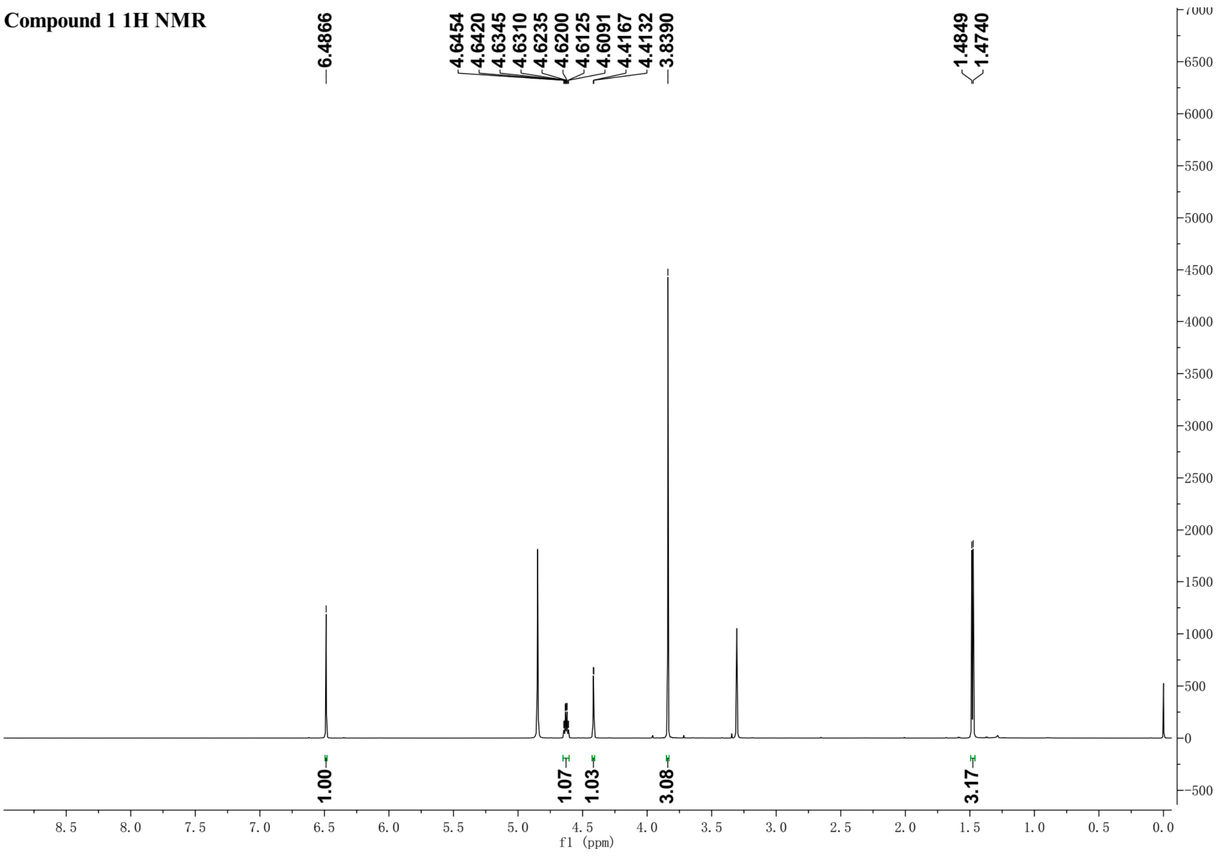

Figure S2. ^{13}C NMR and DEPT135 spectra (125 MHz) of compound **1** in CH_3OH-*d*4

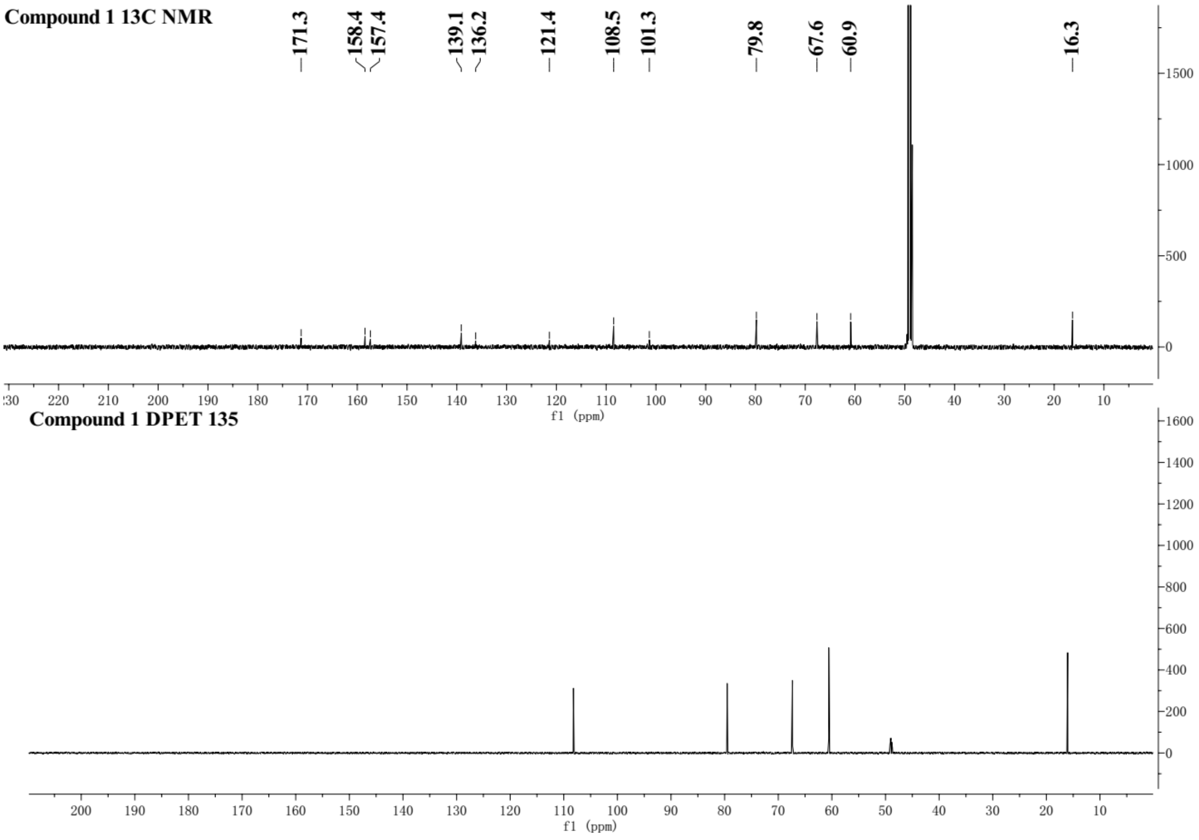

Figure S3. HSQC spectrum (500 MHz) of compound **1** in CH_3OH-d_4

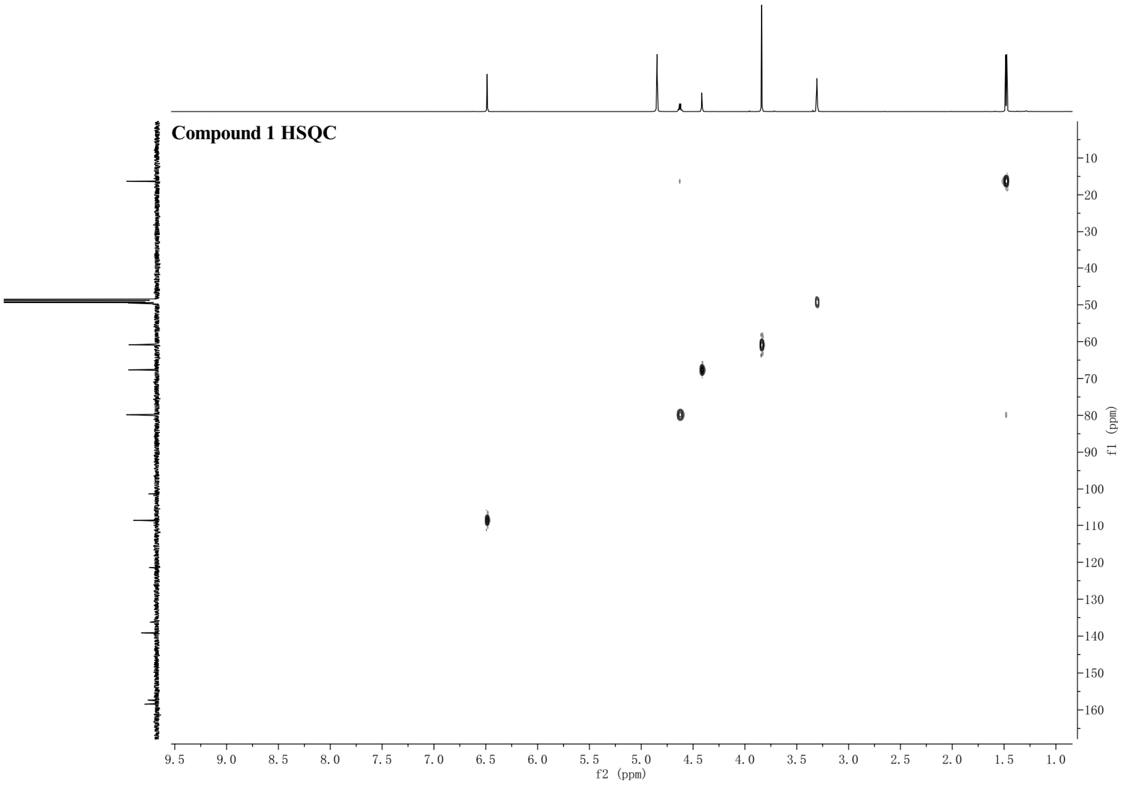

Figure S4. ^1H-^1H COSY spectrum (500 MHz) of compound **1** in CH_3OH-d_4

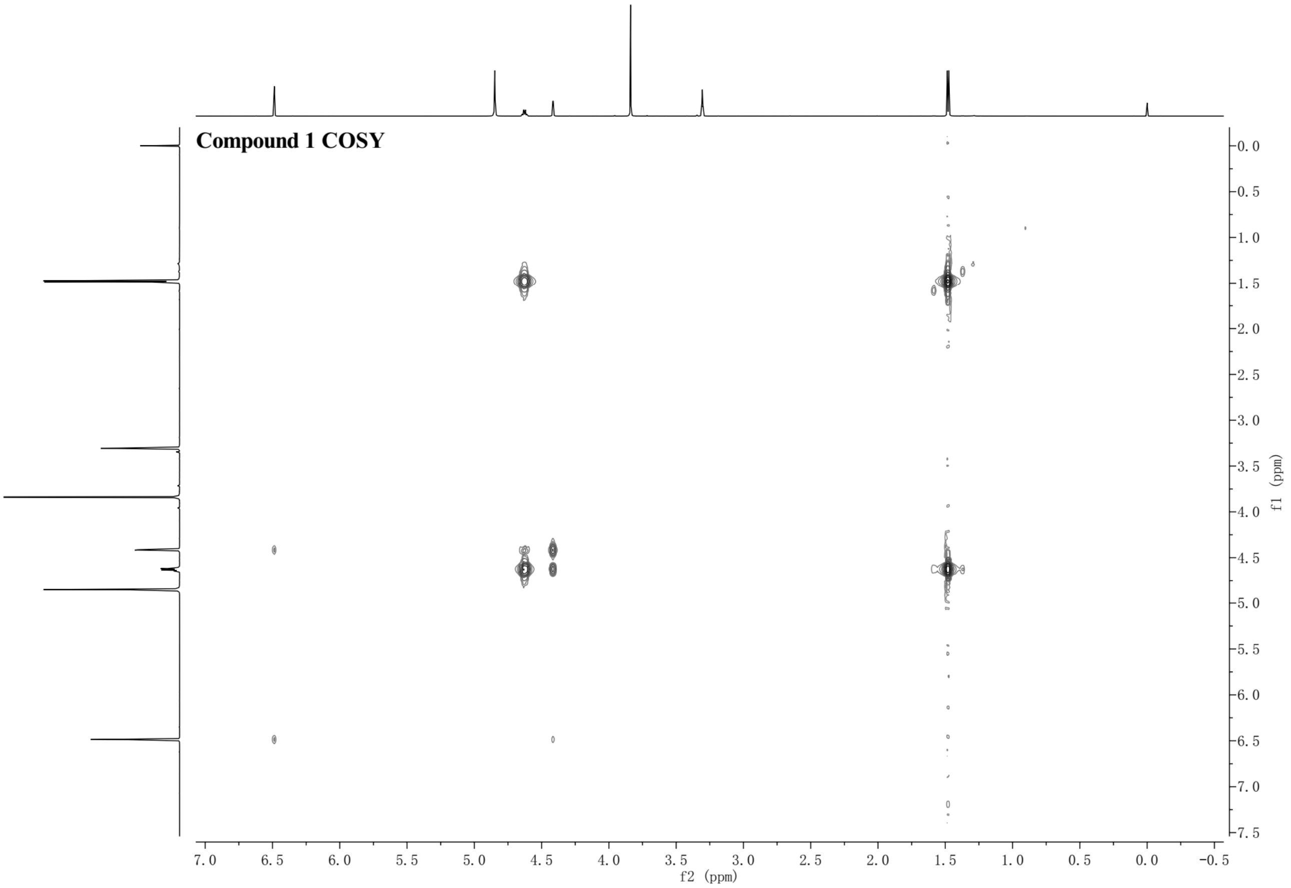

Figure S5. HMBC spectrum (500 MHz) of compound **1** in CH_3OH-d_4

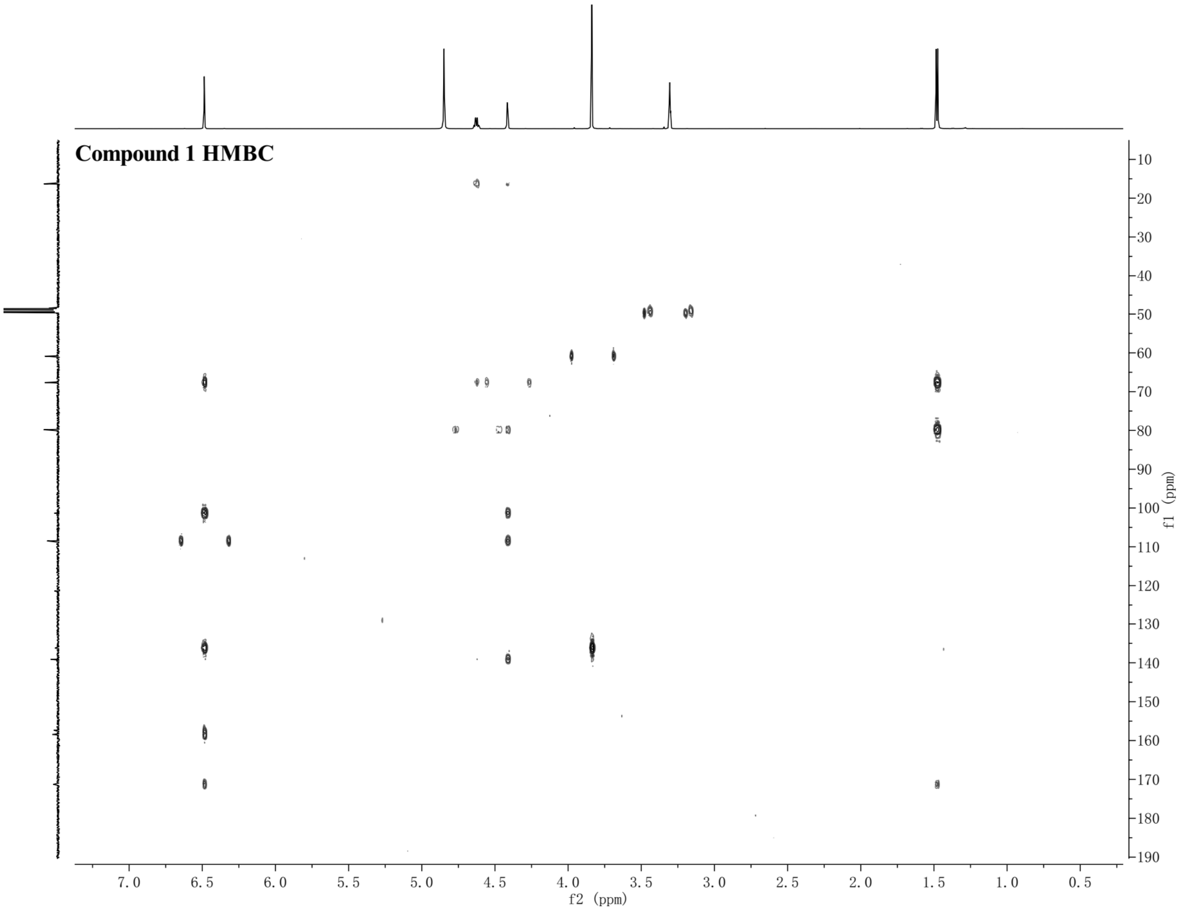

Figure S6. HRESIMS spectrum for compound **1**

Mass Spectrum SmartFormula Report

Analysis Info

Acquisition Date 2021-07-05 23:26:19

Analysis Name	D:\Data\A501\YJZ\2021.7.5\whf-01_02_RA6_01_2573.d		
Method	lc-ms_as_ms.m	Operator	Demo User
Sample Name	whf-01_02	Instrument	compact 8255754.20156
Comment			

Acquisition Parameter

Source Type	ESI	Ion Polarity	Positive	Set Nebulizer	1.8 Bar
Focus	Not active	Set Capillary	4500 V	Set Dry Heater	250 °C
Scan Begin	50 m/z	Set End Plate Offset	-500 V	Set Dry Gas	4.0 l/min
Scan End	2500 m/z	Set Charging Voltage	2000 V	Set Divert Valve	Waste
		Set Corona	0 nA	Set APCI Heater	0 °C

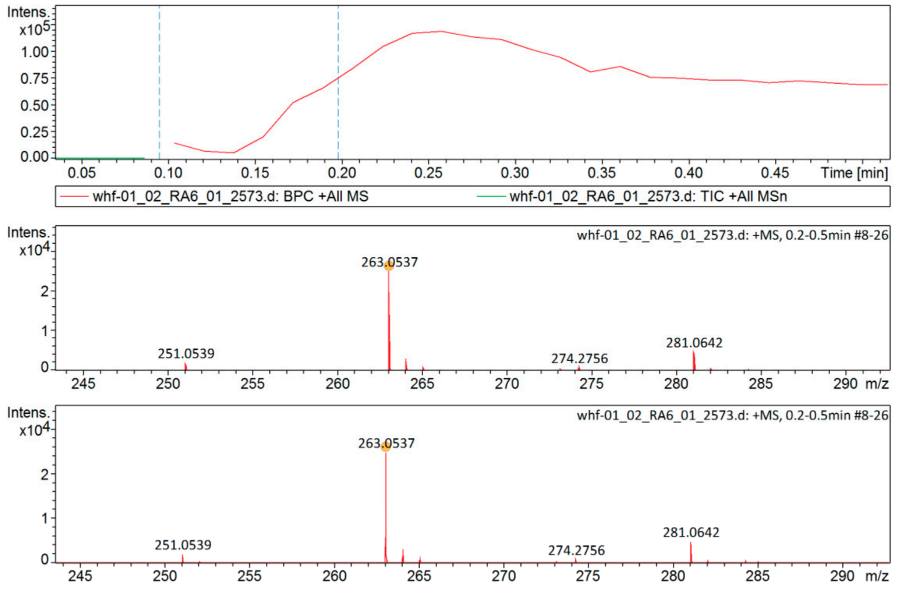

Meas. m/z	#	Ion Formula	m/z	err [ppm]	mSigma	# mSigma	Score	rdb	e⁻ Conf	N-Rule	Adduct
263.0537	1	C11H12NaO6	263.0526	-4.2	12.8	1	100.00	6.0	even	ok	M+Na

whf-01_02_RA6_01_2573.d
Bruker Compass DataAnalysis 4.4 printed: 2021-07-07 17:23:04 by: demo Page 1 of 1

Figure S7. ^{1}H NMR spectrum (500 MHz) data for compound **2** in CH_3OH-d_4

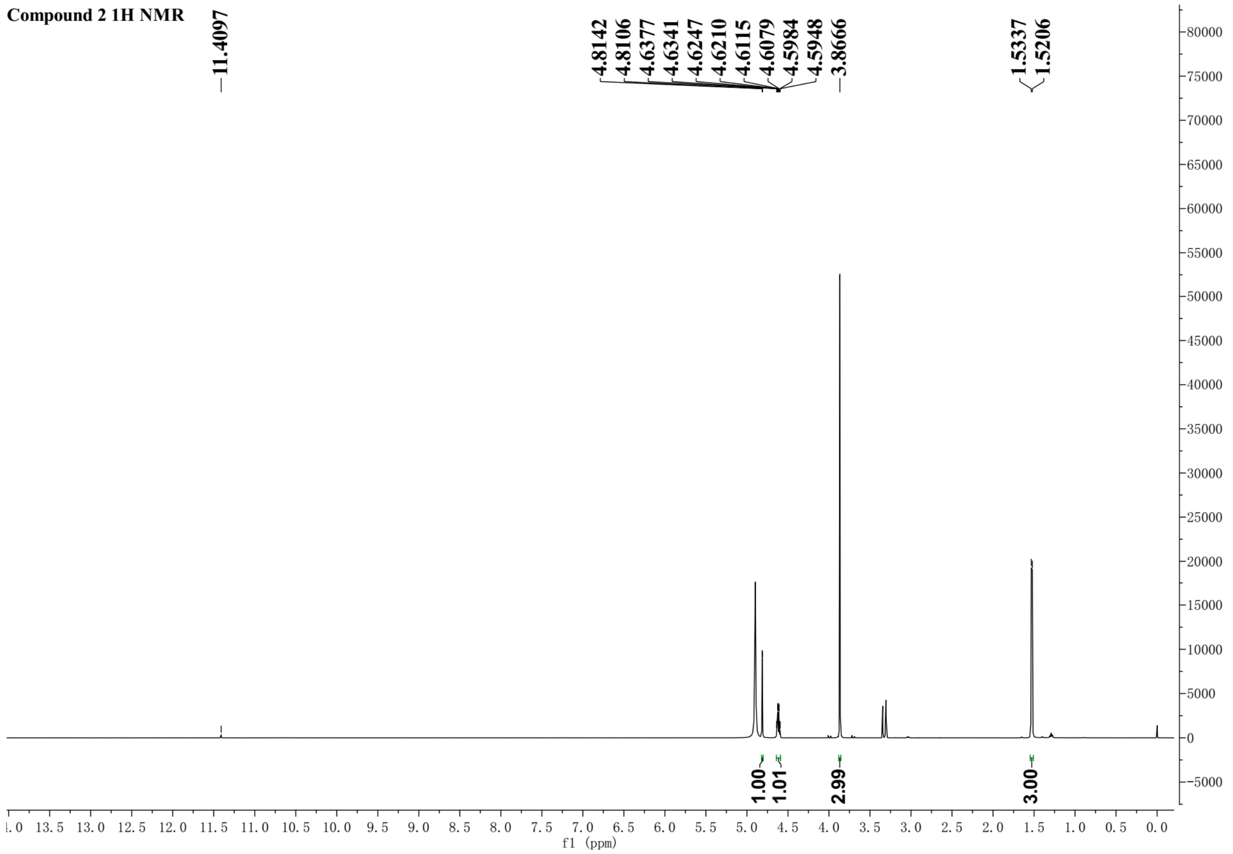

Figure S8. ^{13}C NMR and DEPT135 spectra (125 MHz) data for compound **2** in CH_3OH-d_4

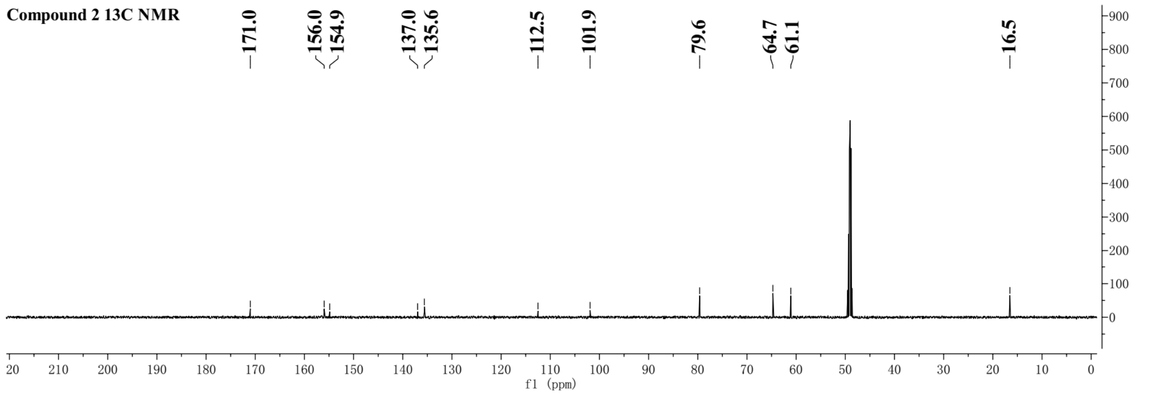

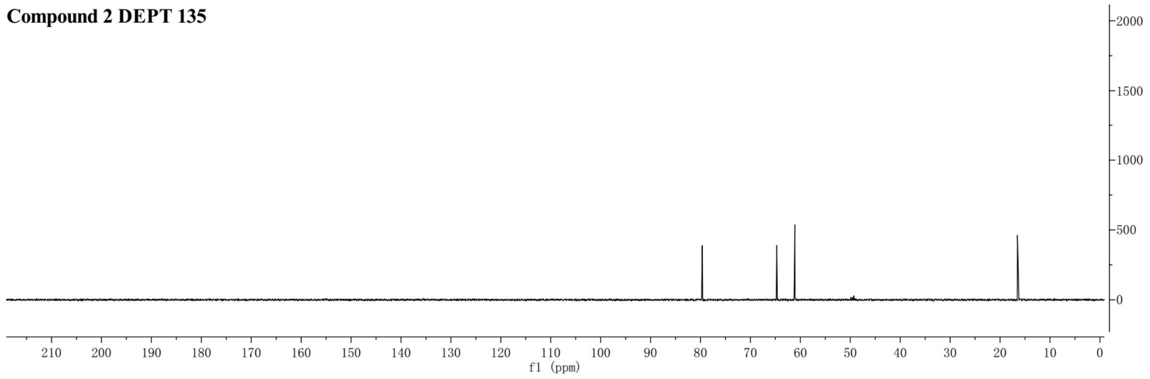

Figure S9. HSQC spectrum (500 MHz) of compound **2** in CH_3OH-*d_4*

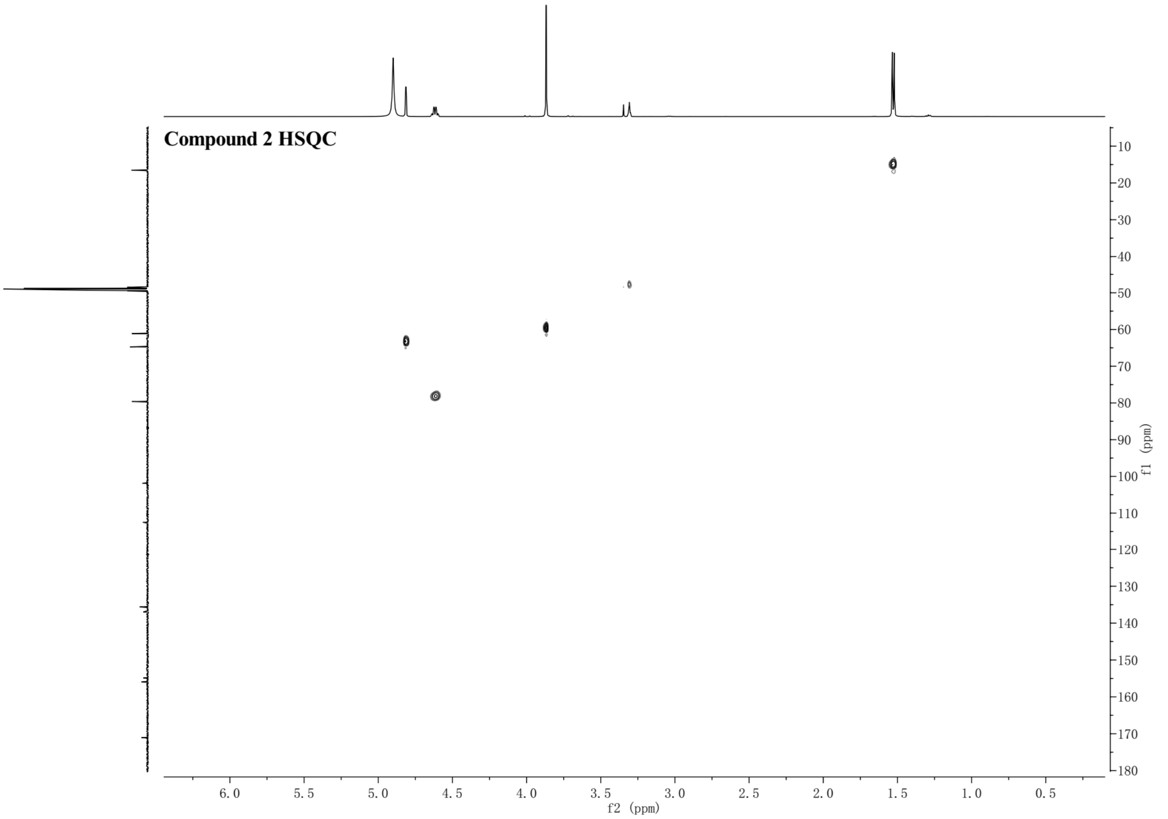

Figure S10. ^1H-^1H COSY spectrum (500 MHz) of compound **2** in CH_3OH-*d_4*

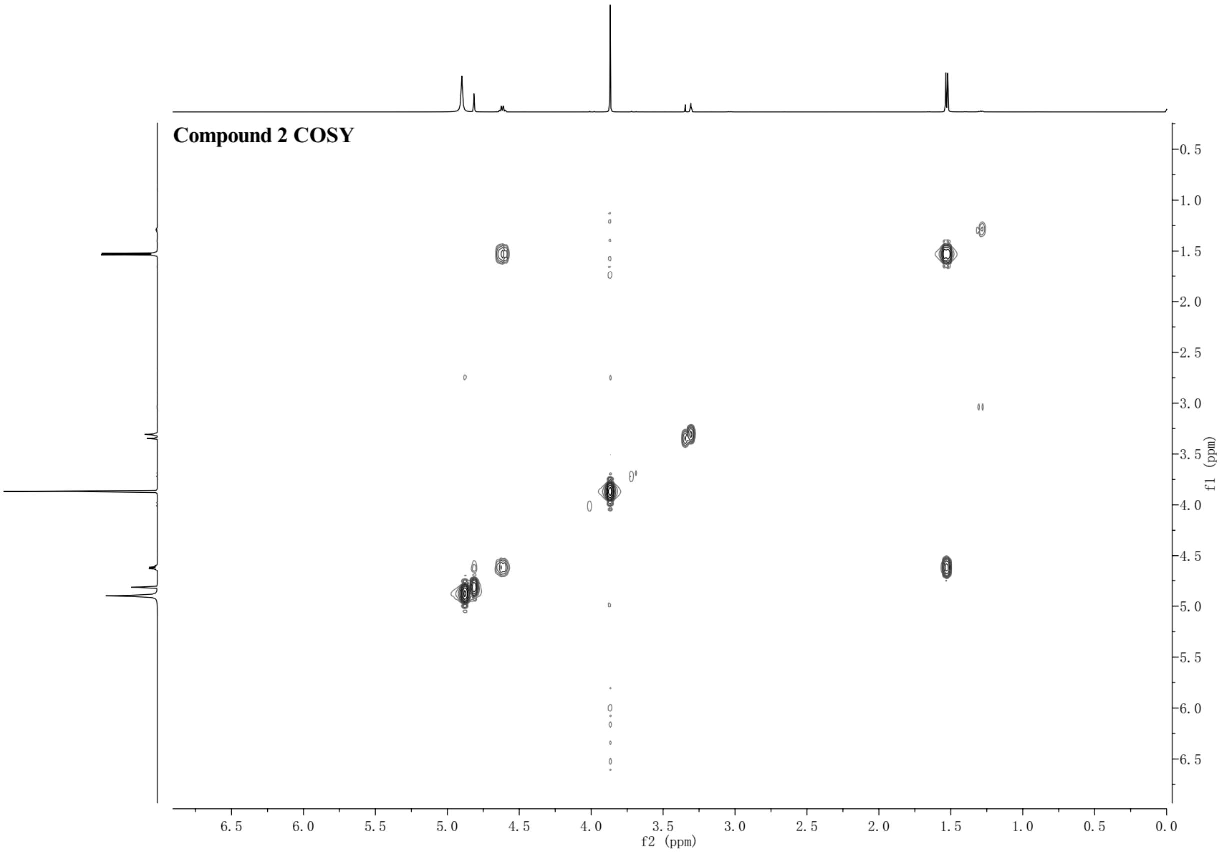

Figure S11. HMBC spectrum (500 MHz) of compound **2** in CH_3OH-d_4

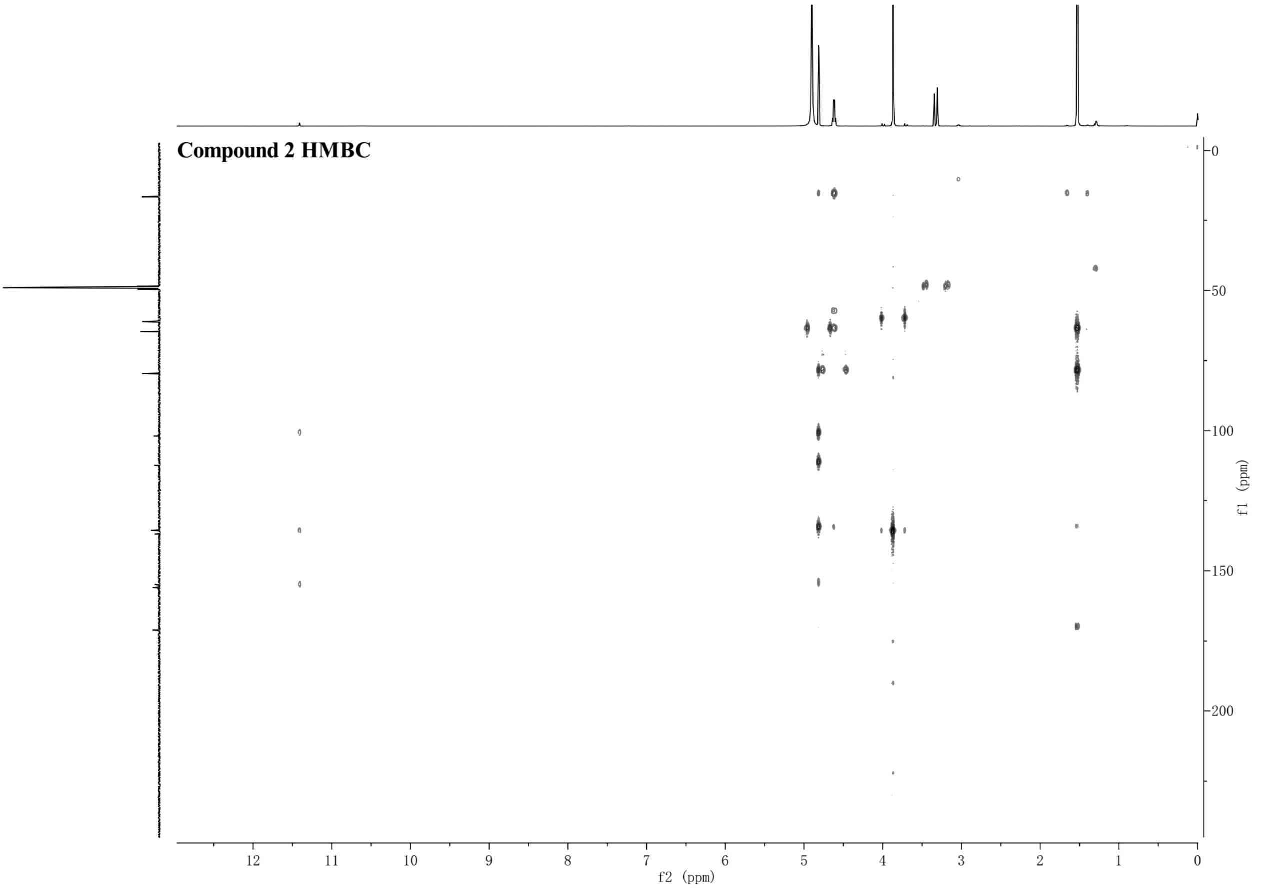

Figure S12. HRESIMS spectrum for compound **2**

Mass Spectrum SmartFormula Report

Analysis Info

Analysis Name	D:\Data\A501\YJZ\2021.7.5\whf-26-1_RA1_01_2568.d	Acquisition Date	2021-07-05 23:12:10
Method	lc-ms_as_ms.m	Operator	Demo User
Sample Name	whf-26-1	Instrument	compact 8255754.20156
Comment			

Acquisition Parameter

Source Type	ESI	Ion Polarity	Positive	Set Nebulizer	1.8 Bar
Focus	Not active	Set Capillary	4500 V	Set Dry Heater	250 °C
Scan Begin	50 m/z	Set End Plate Offset	-500 V	Set Dry Gas	4.0 l/min
Scan End	2500 m/z	Set Charging Voltage	2000 V	Set Divert Valve	Waste
		Set Corona	0 nA	Set APCI Heater	0 °C

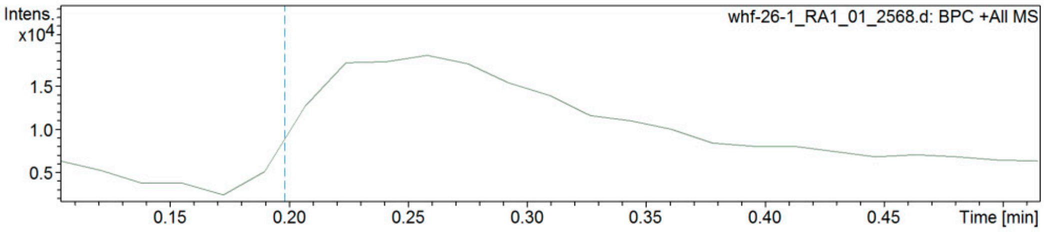

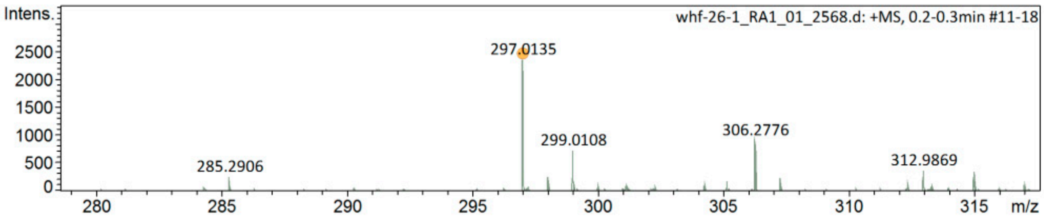

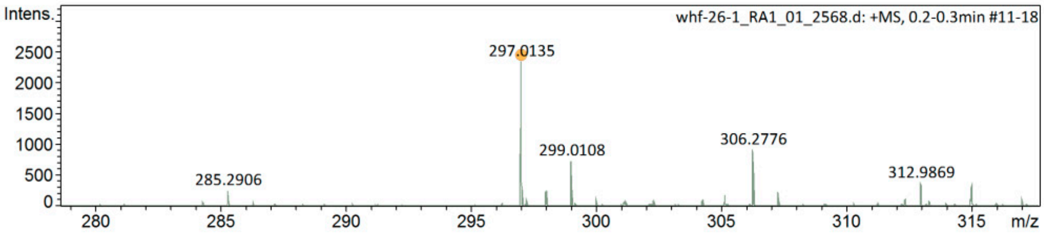

Meas. m/z	#	Ion Formula	m/z	err [ppm]	mSigma	# mSigma	Score	rdb	e¯ Conf	N-Rule	Adduct
297.0135	1	C11H11ClNaO6	297.0136	0.6	26.4	1	100.00	6.0	even	ok	M+Na

whf-26-1_RA1_01_2568.d

Bruker Compass DataAnalysis 4.4 printed: 2022-06-14 16:39:30 by: demo Page 1 of 1

Figure S13. ^{1}H NMR spectrum (500 MHz) data for compound **3** in CH_3OH-d_4

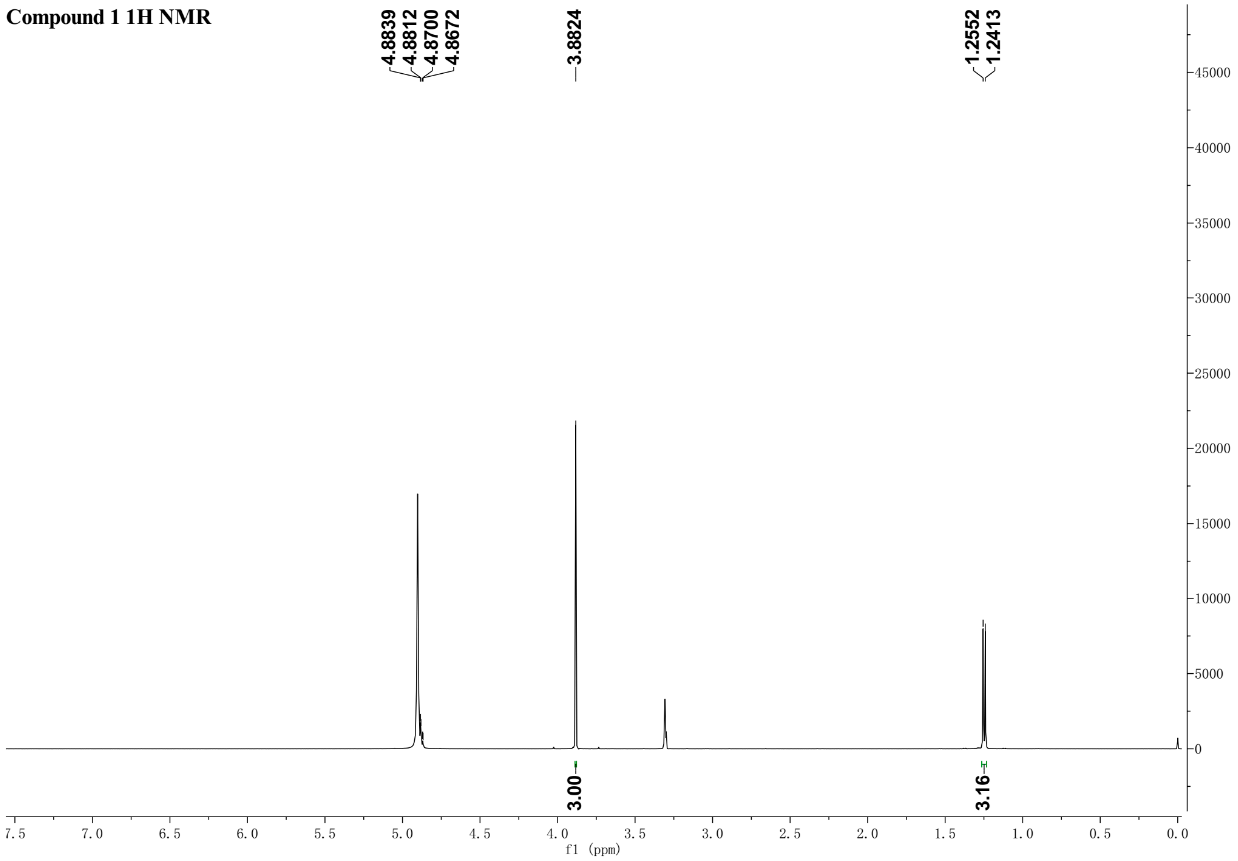

Figure S14. ^{13}C NMR and DEPT135 spectra (125 MHz) data for compound **3** in CH_3OH-d_4

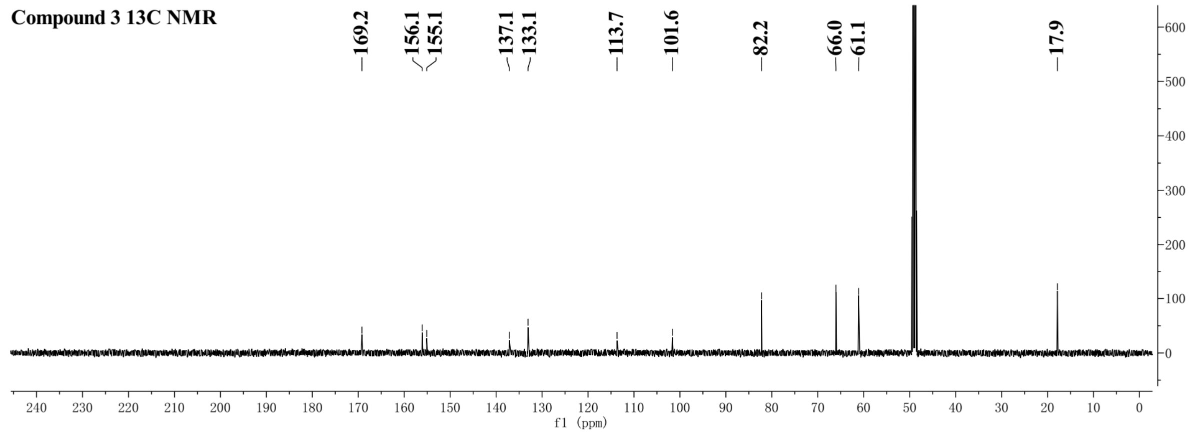

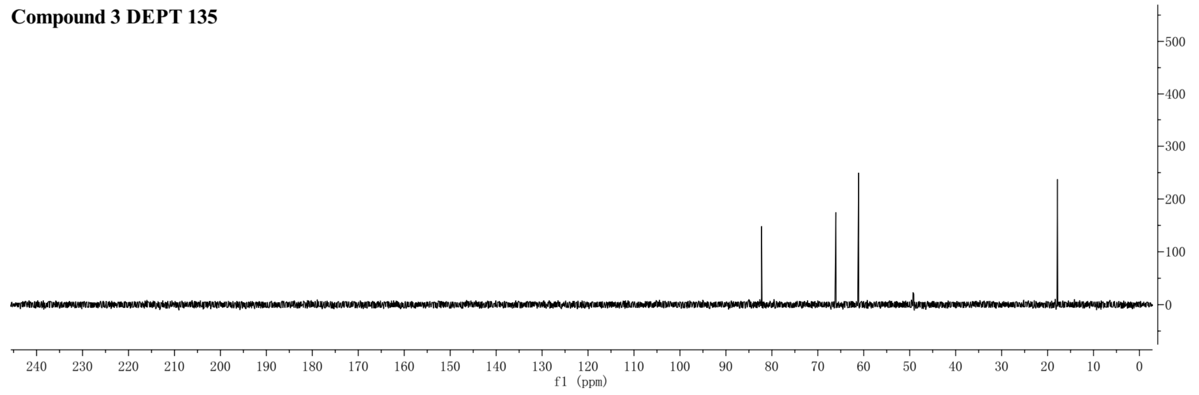

Figure S15. HSQC spectrum (500 MHz) of compound **3** in CH_3OH-d_4

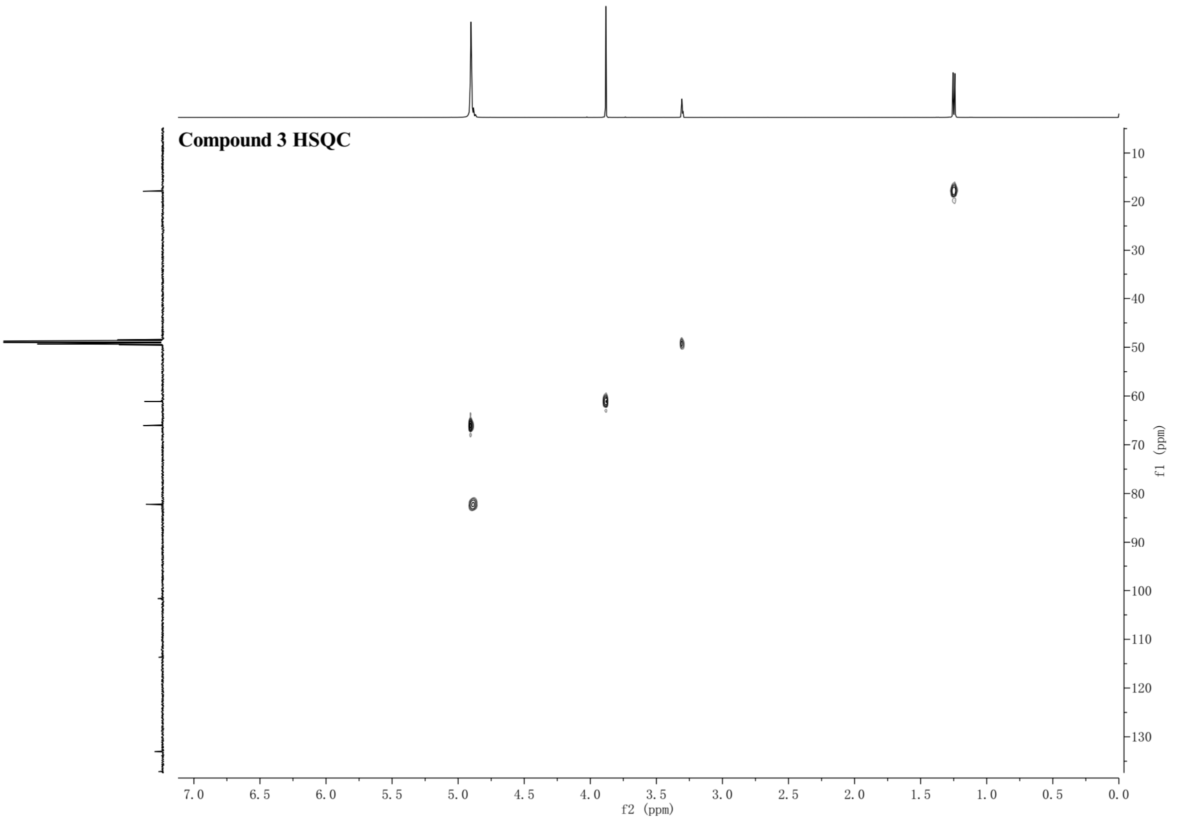

Figure S16. ^1H-^1H COSY spectrum (500 MHz) of compound **3** in CH_3OH-d_4

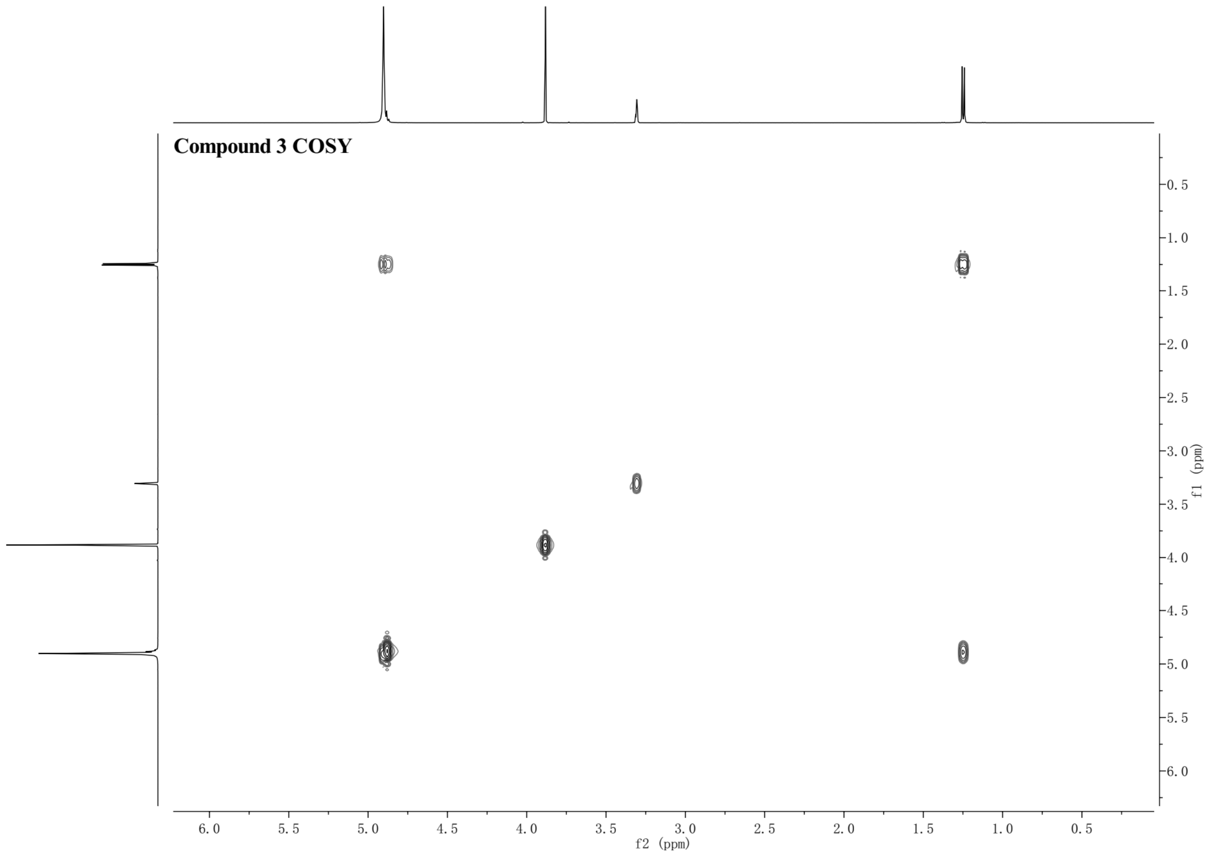

Figure S17. HMBC spectrum (500 MHz) of compound **3** in CH_3OH-d_4

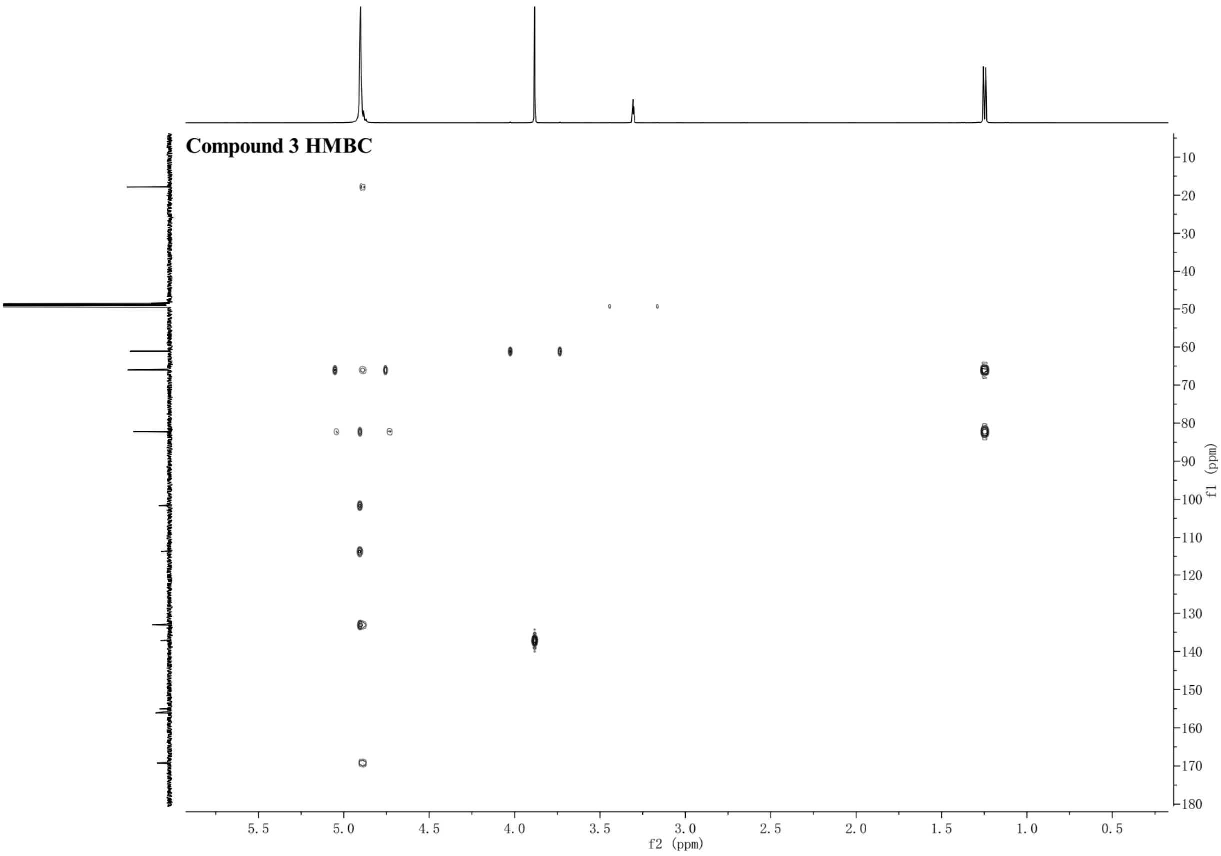

Figure S18. ROESY spectrum (500 MHz) of compound **3** in CH_3OH-d_4

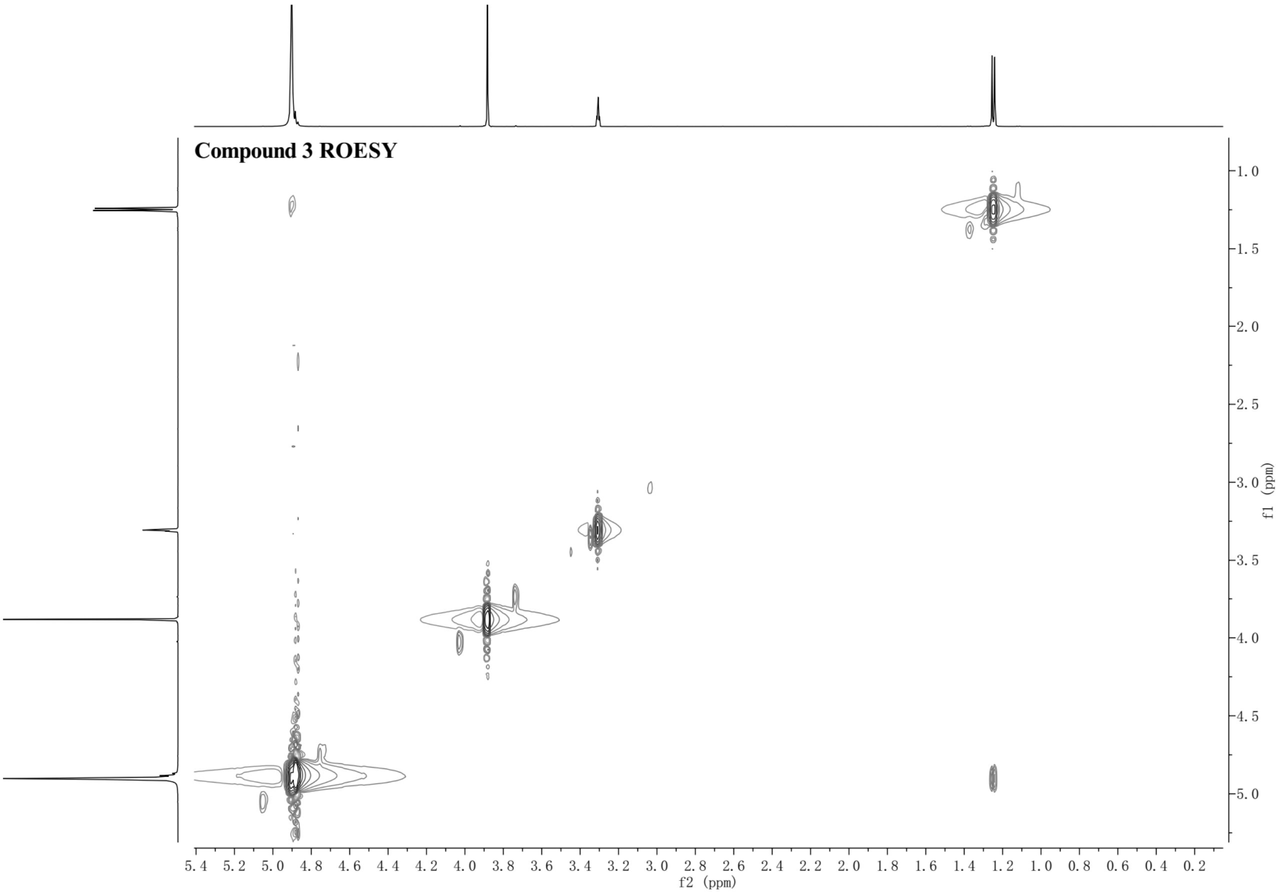

Figure S19. 1D NOESY spectrum (500 MHz) of compound **3** in CH_3OH-*d_4*

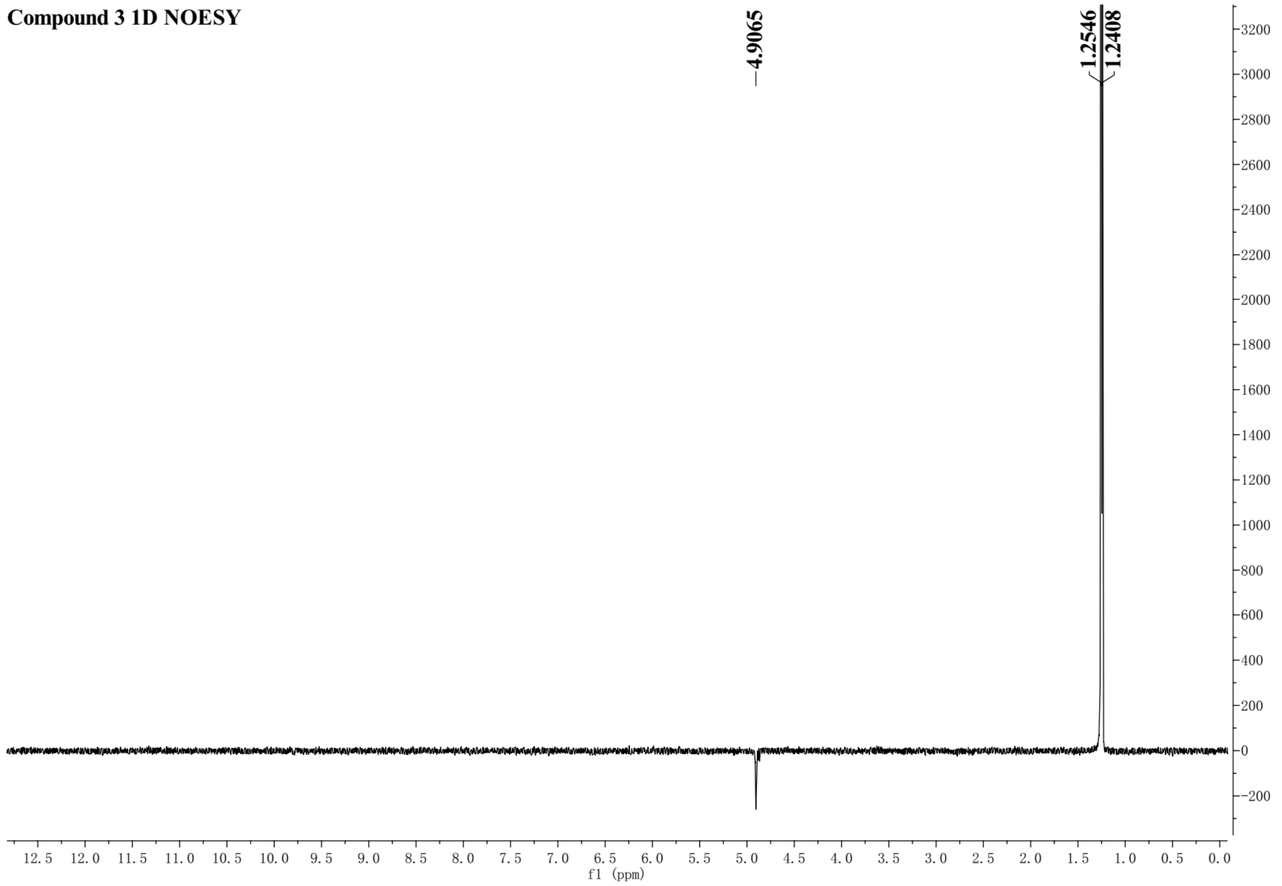

Figure S 20. HRESIMS spectrum for compound **3**

Mass Spectrum SmartFormula Report

Analysis Info

Analysis Name	D:\Data\A501\YJZ\2021.7.5\whf-26-02_RA3_01_2570.d	Acquisition Date	2021-07-05 23:17:50
Method	lc-ms_as_ms.m	Operator	Demo User
Sample Name	whf-26-02	Instrument	compact 8255754.20156
Comment			

Acquisition Parameter

Source Type	ESI	Ion Polarity	Positive	Set Nebulizer	1.8 Bar
Focus	Not active	Set Capillary	4500 V	Set Dry Heater	250 °C
Scan Begin	50 m/z	Set End Plate Offset	-500 V	Set Dry Gas	4.0 l/min
Scan End	2500 m/z	Set Charging Voltage	2000 V	Set Divert Valve	Waste
		Set Corona	0 nA	Set APCI Heater	0 °C

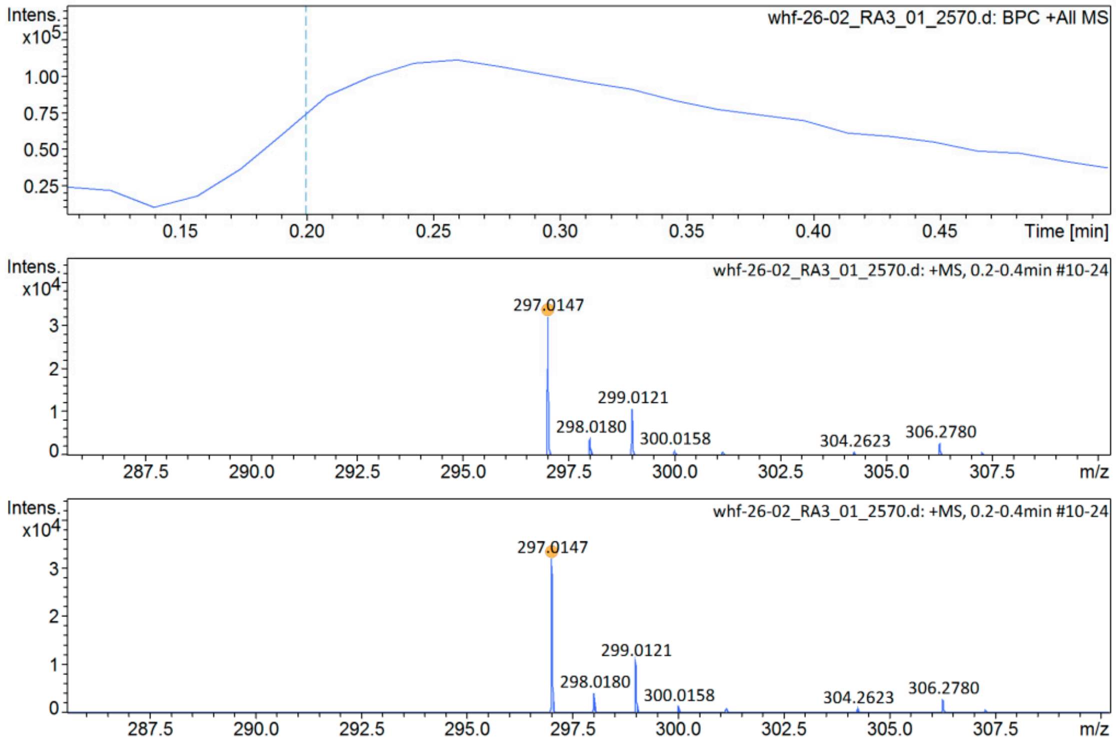

Meas. m/z	#	Ion Formula	m/z	err [ppm]	mSigma	# mSigma	Score	rdb	e⁻ Conf	N-Rule	Adduct
297.0147	1	C11H11ClNaO6	297.0136	-3.4	4.4	1	100.00	6.0	even	ok	M+Na

Figure S21. ^{1}H NMR spectrum (600 MHz) data for compound **4** in CH_3OH-*d*4

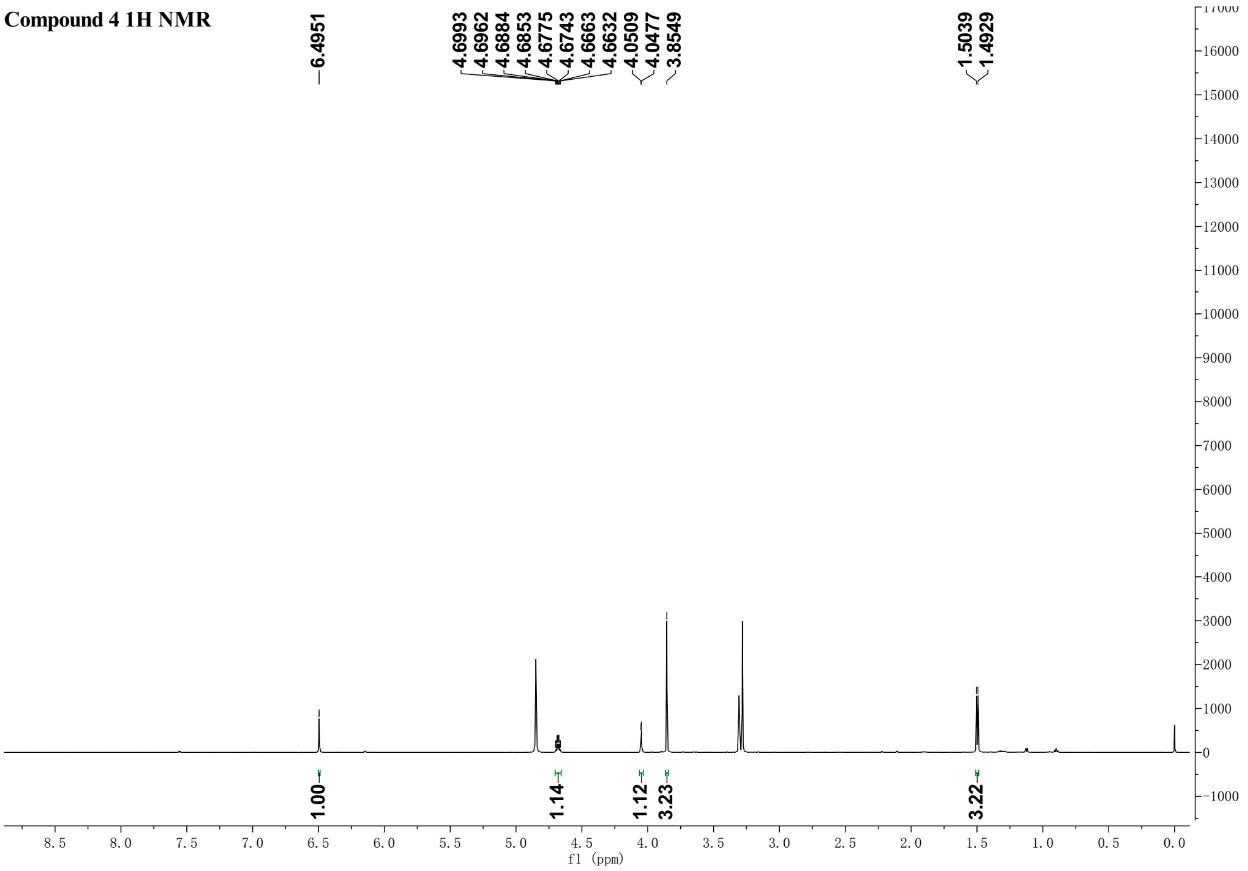

Figure S22. ^{13}C NMR and DEPT135 spectra (150 MHz) data for compound **4** in CH_3OH-*d*4

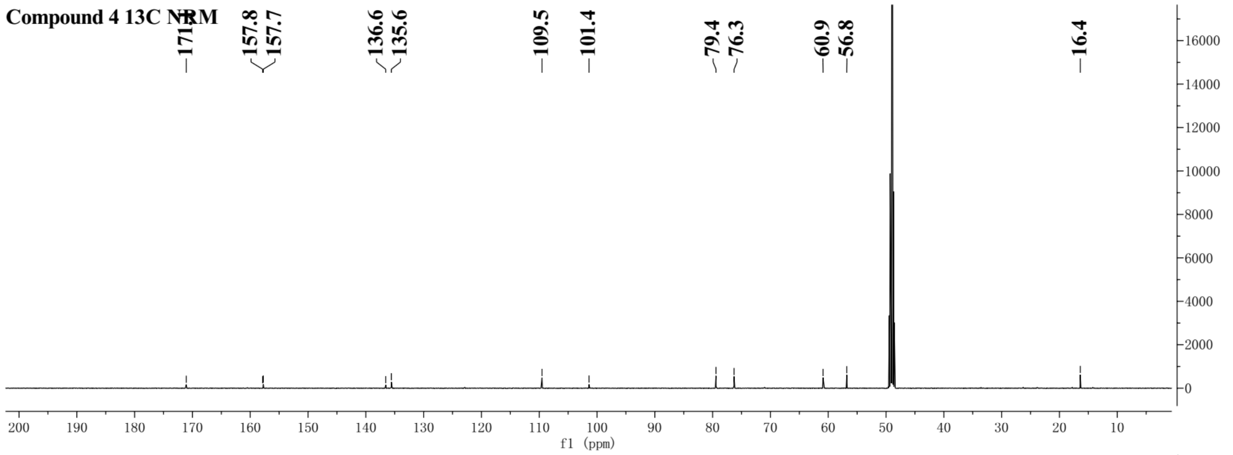

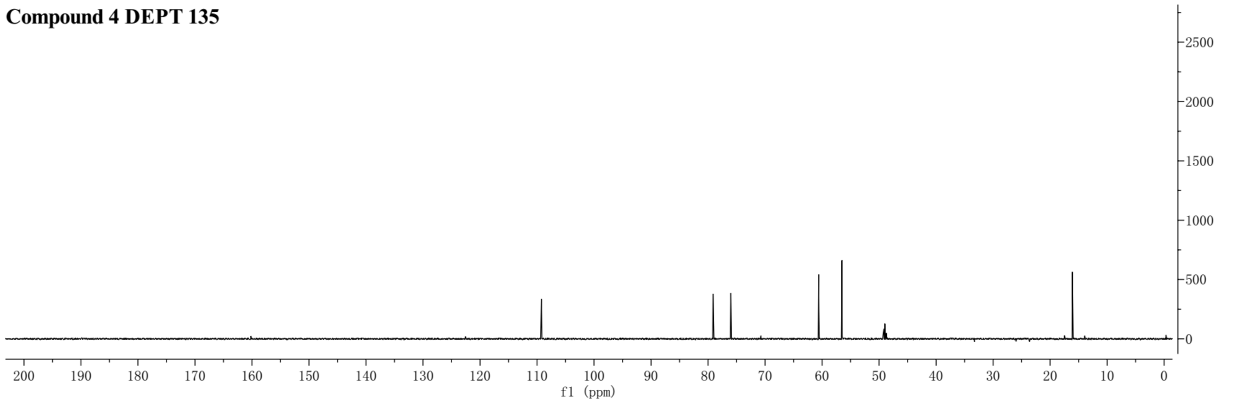

Figure S23. HSQC spectrum (600 MHz) of compound **4** in CH_3OH-d_4

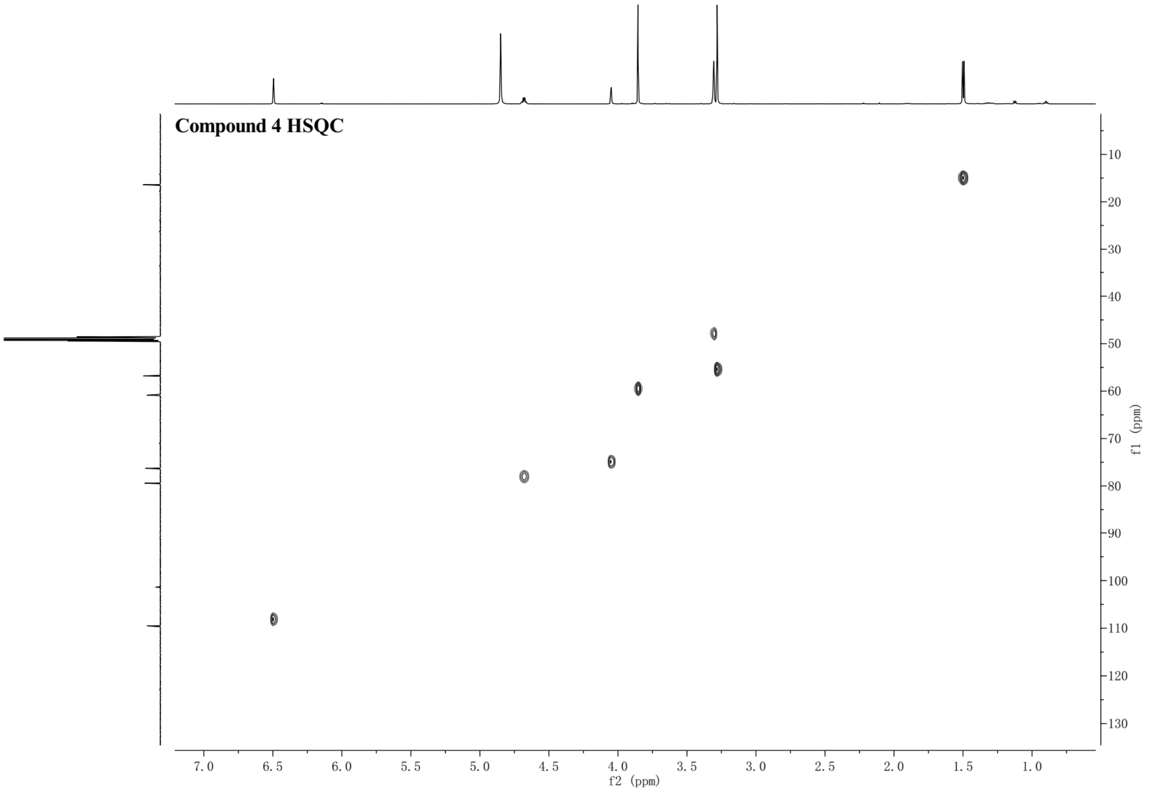

Figure S24. ^1H-^1H COSY spectrum (600 MHz) of compound **4** in CH_3OH-d_4

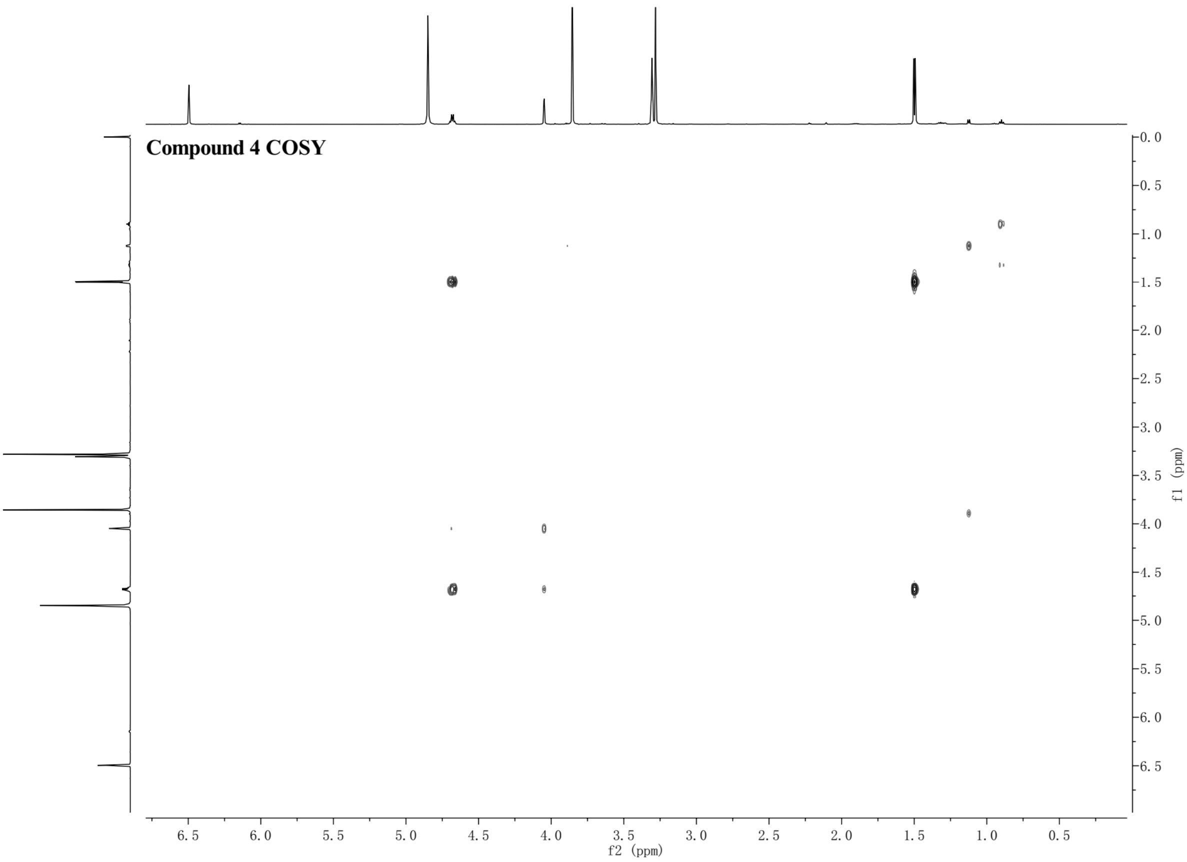

Figure S25. HMBC spectrum (600 MHz) of compound **4** in CH_3OH-d_4

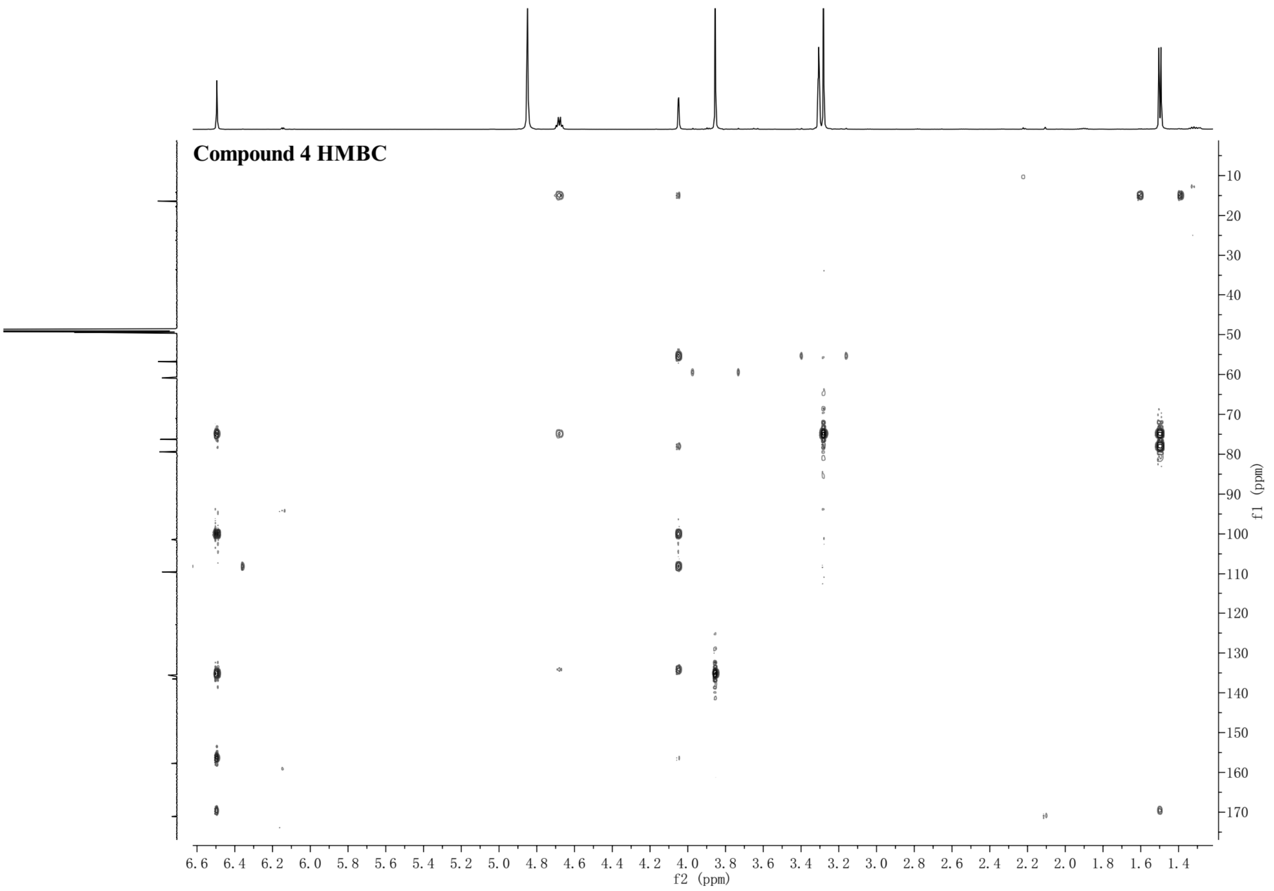

Figure S26. HRESIMS spectrum for compound **4**

Mass Spectrum SmartFormula Report

Analysis Info

Analysis Name	D:\Data\A501\YJZ\2021.11.1\WHF-24_BC3_01_3186.d	Acquisition Date	2021-11-01 22:06:13	
Method	lc-ms_as_ms.m	Operator	Demo User	
Sample Name	WHF-24	Instrument	compact	8255754.20156
Comment				

Acquisition Parameter

Source Type	ESI	Ion Polarity	Positive	Set Nebulizer	1.8 Bar
Focus	Not active	Set Capillary	4500 V	Set Dry Heater	220 °C
Scan Begin	50 m/z	Set End Plate Offset	-500 V	Set Dry Gas	4.0 l/min
Scan End	2500 m/z	Set Charging Voltage	2000 V	Set Divert Valve	Waste
		Set Corona	0 nA	Set APCI Heater	0 °C

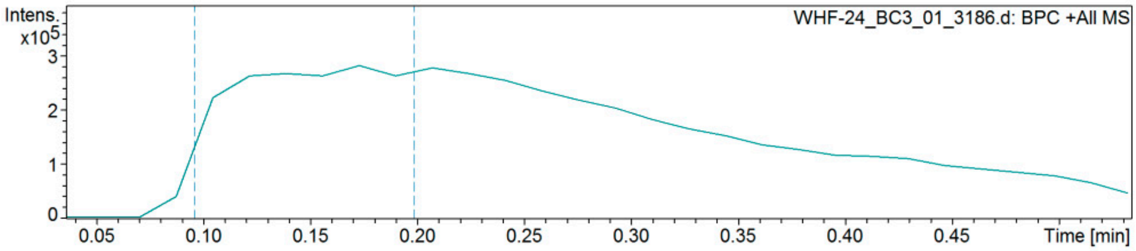

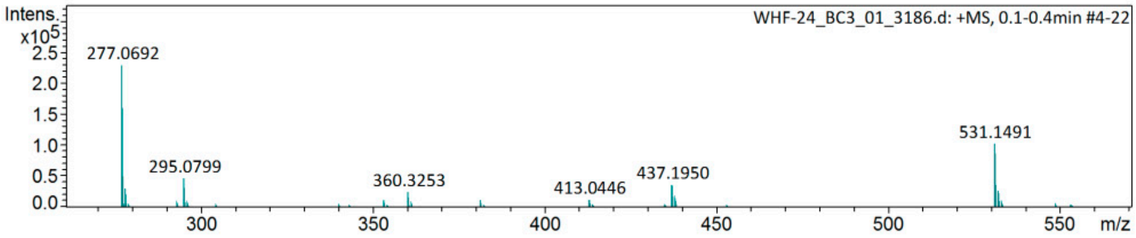

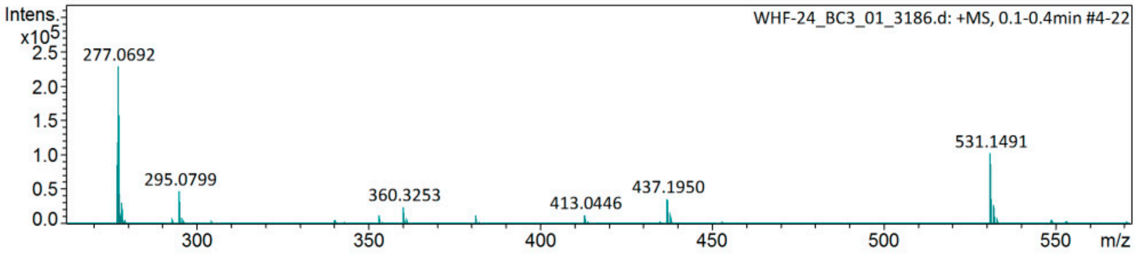

Figure S27. ^{1}H NMR spectrum (600 MHz) data for compound **5** in CH_3OH-*d*$_4$

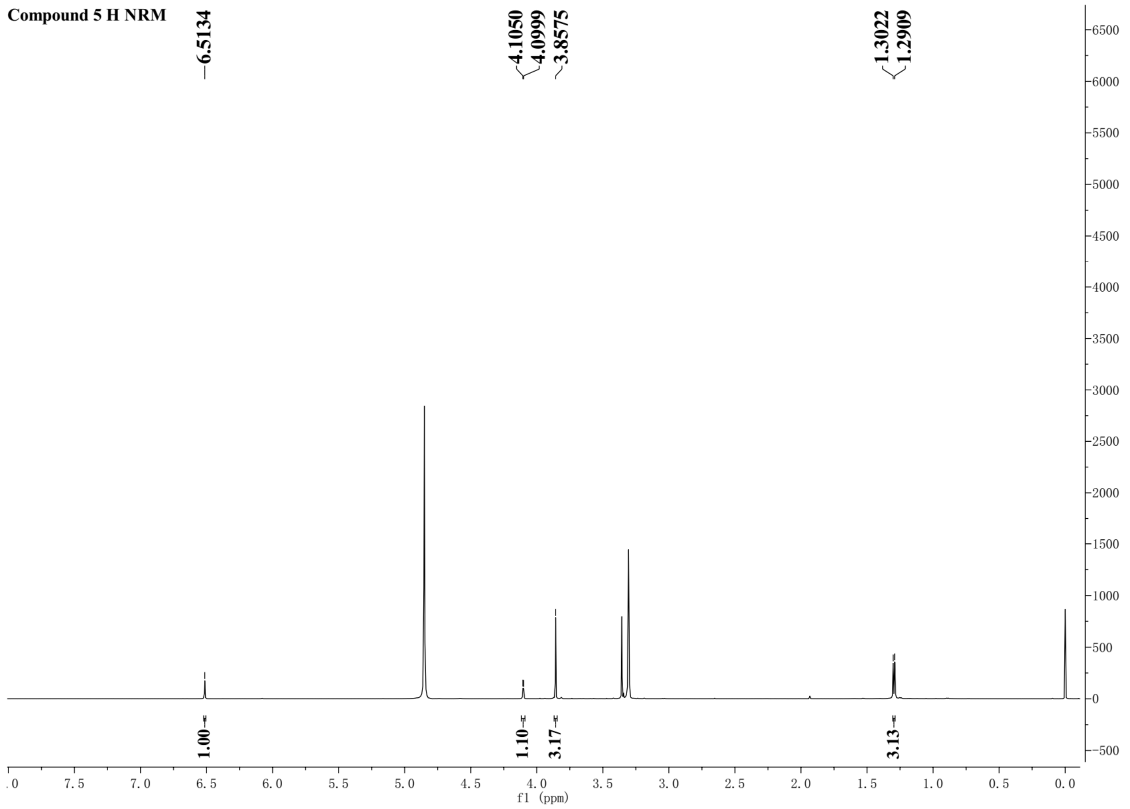

Figure S28. ^{13}C NMR and DEPT135 spectra (150 MHz) data for compound **5** in CH_3OH-*d*$_4$

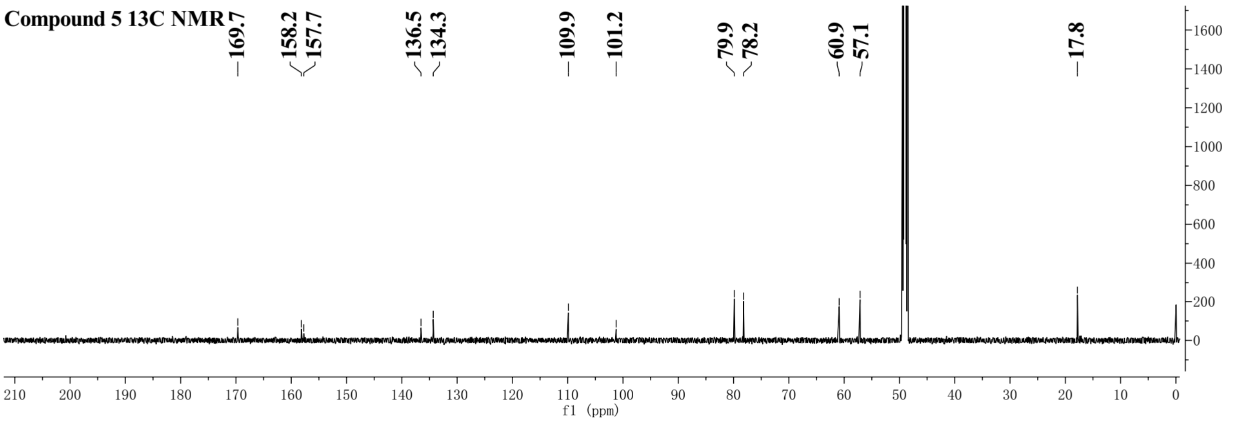

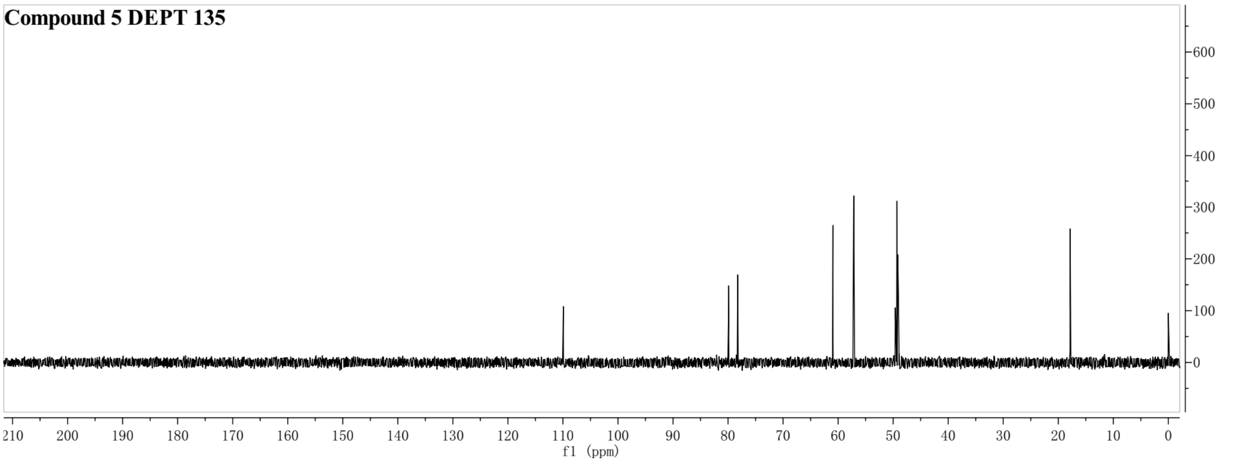

Figure S29. HSQC spectrum (600 MHz) of compound **5** in CH_3OH-d_4

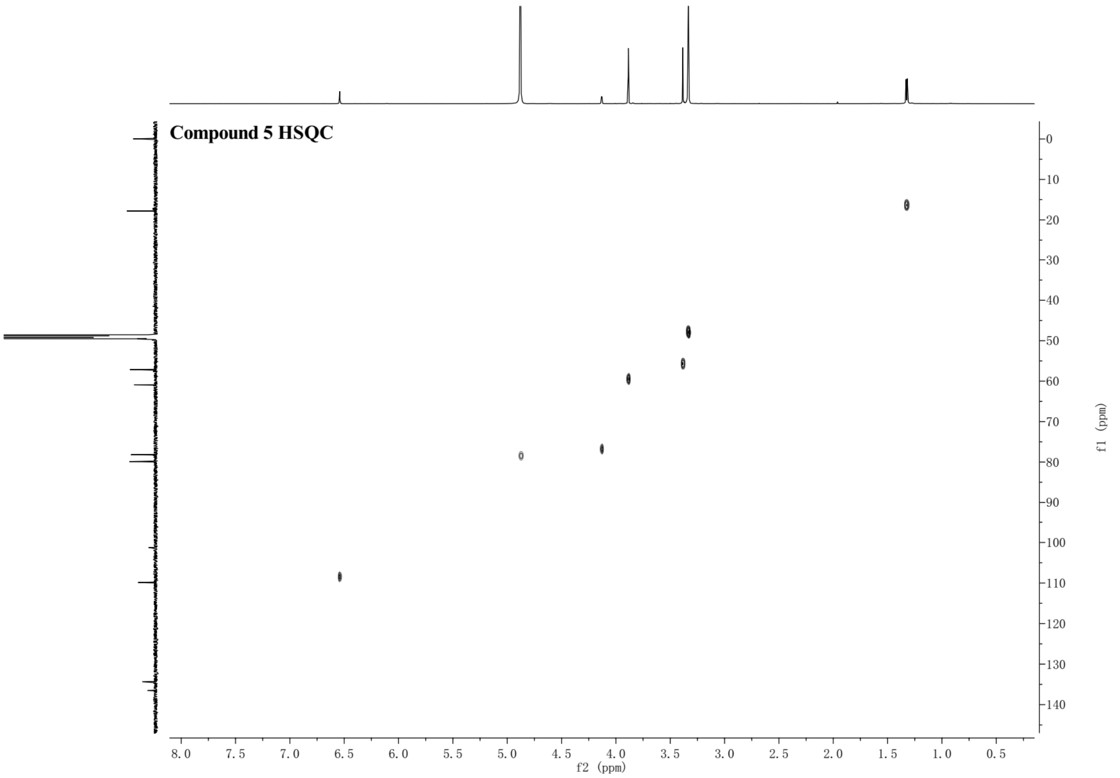

Figure S30. ^1H-^1H COSY spectrum (600 MHz) of compound **5** in CH_3OH-d_4

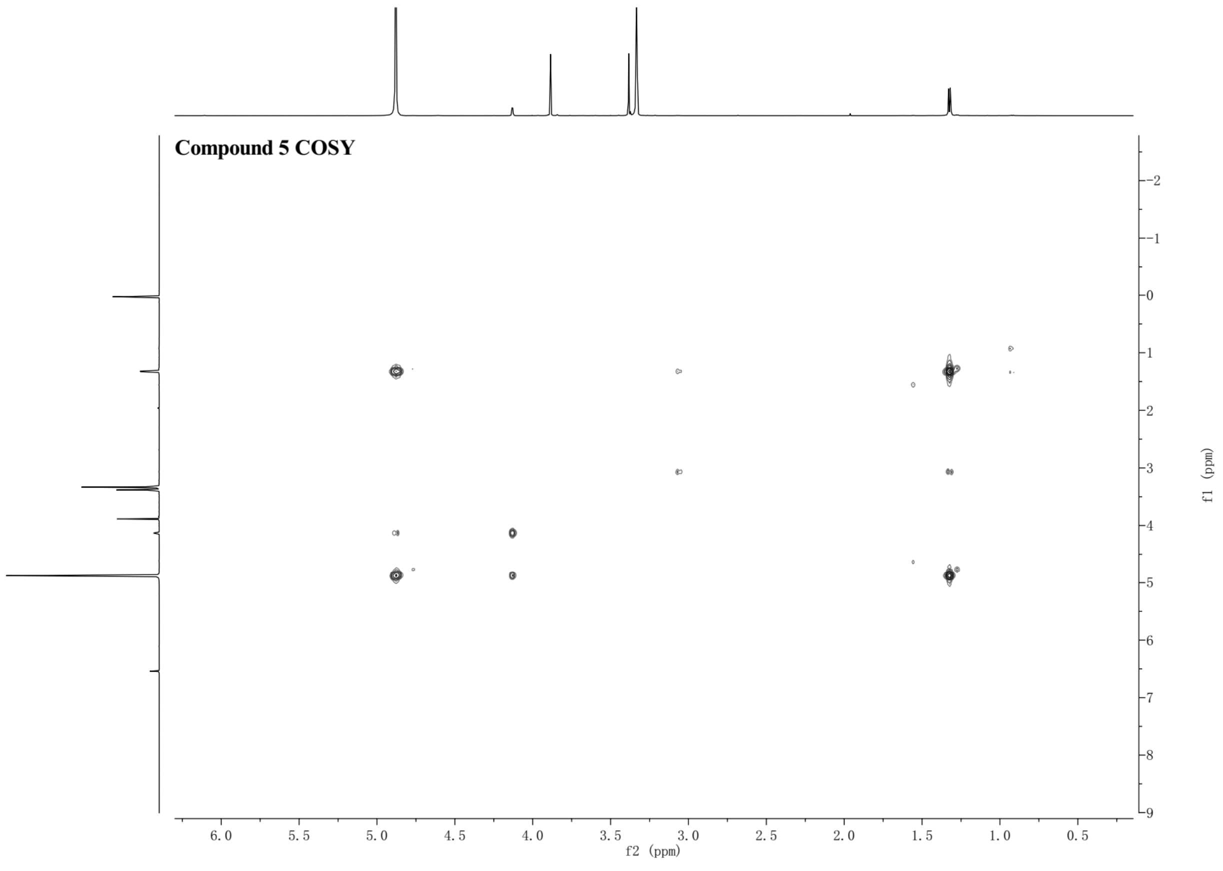

Figure S31. HMBC spectrum (600 MHz) of compound **5** in CH_3OH-d_4

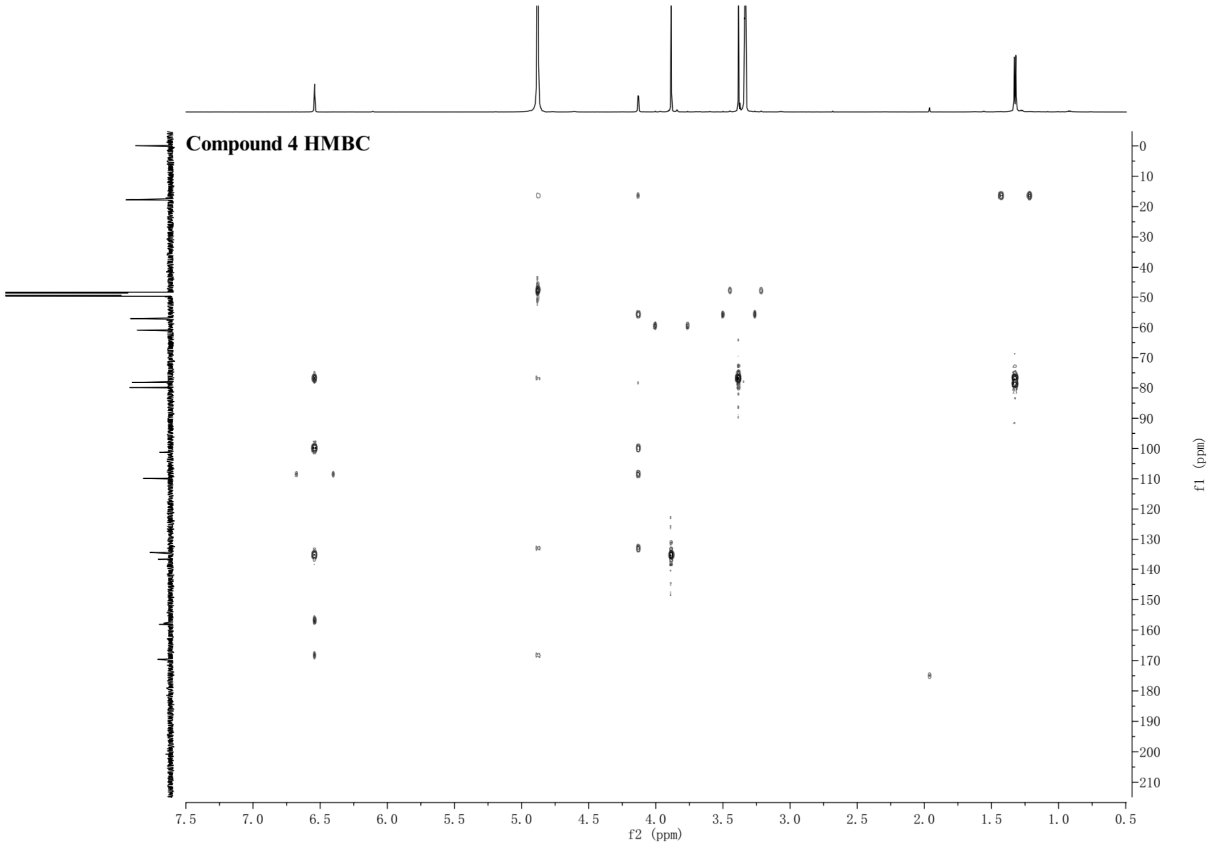

Figure S32. ROESY spectrum (500 MHz) of compound **5** in CH_3OH-d_4

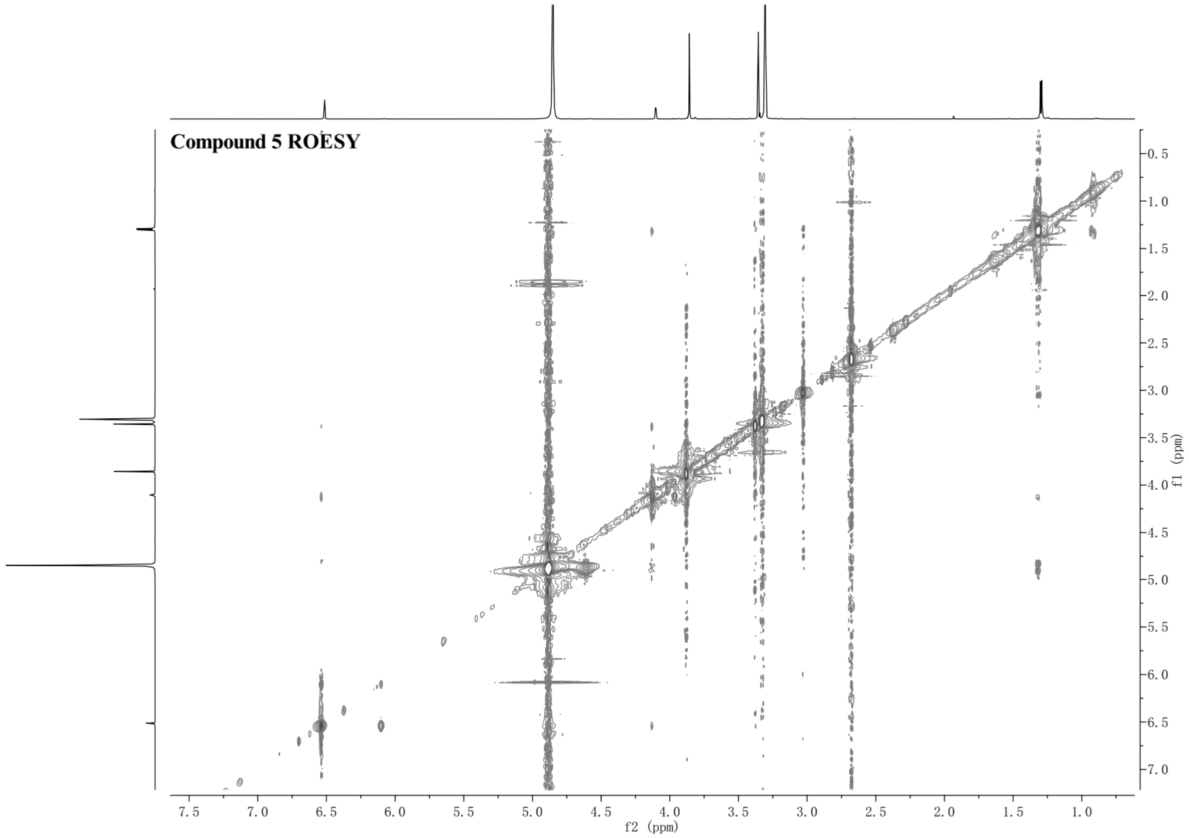

Figure S33. 1D NOESY spectrum (500 MHz) of compound **5** in CH_3OH-d_4

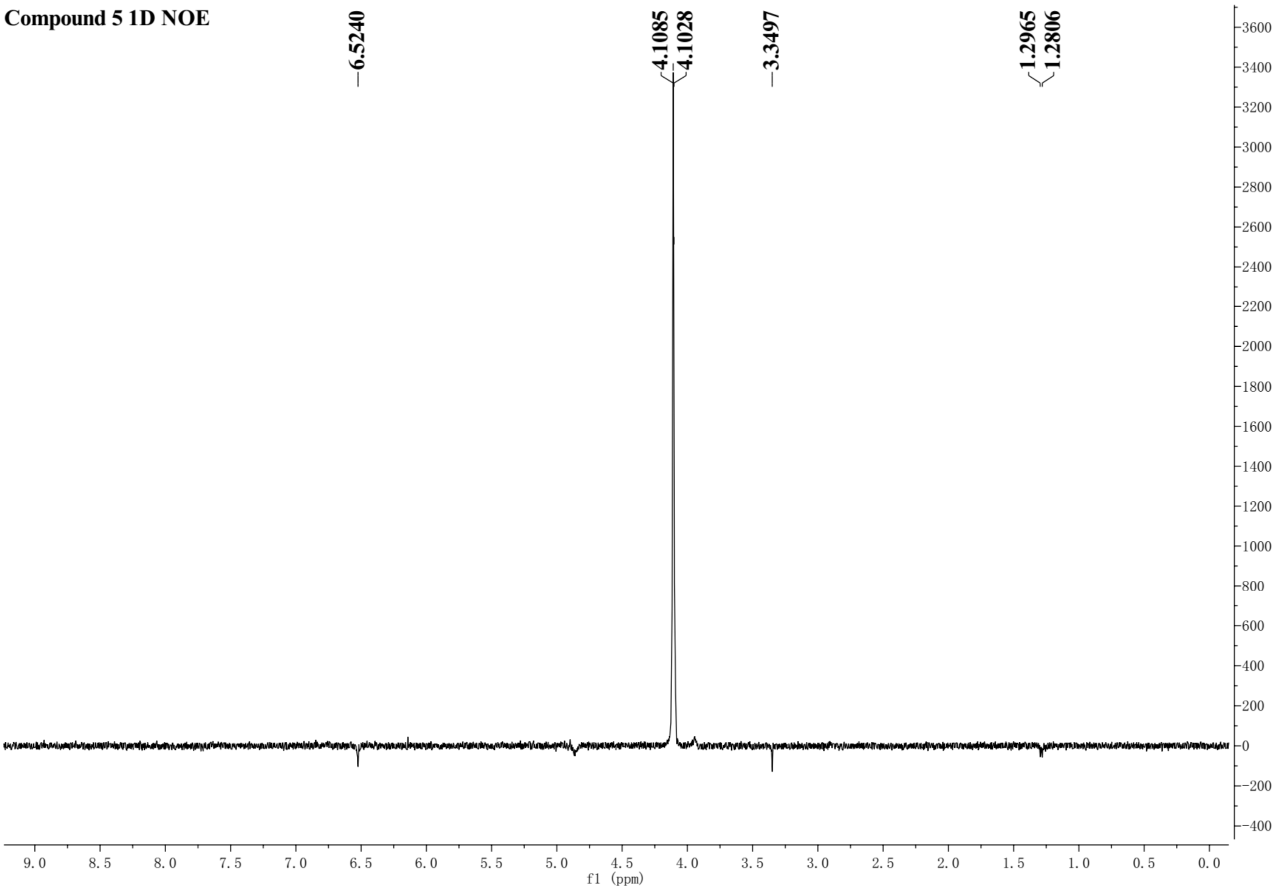

Figure S34. HRESIMS spectrum for compound **5**

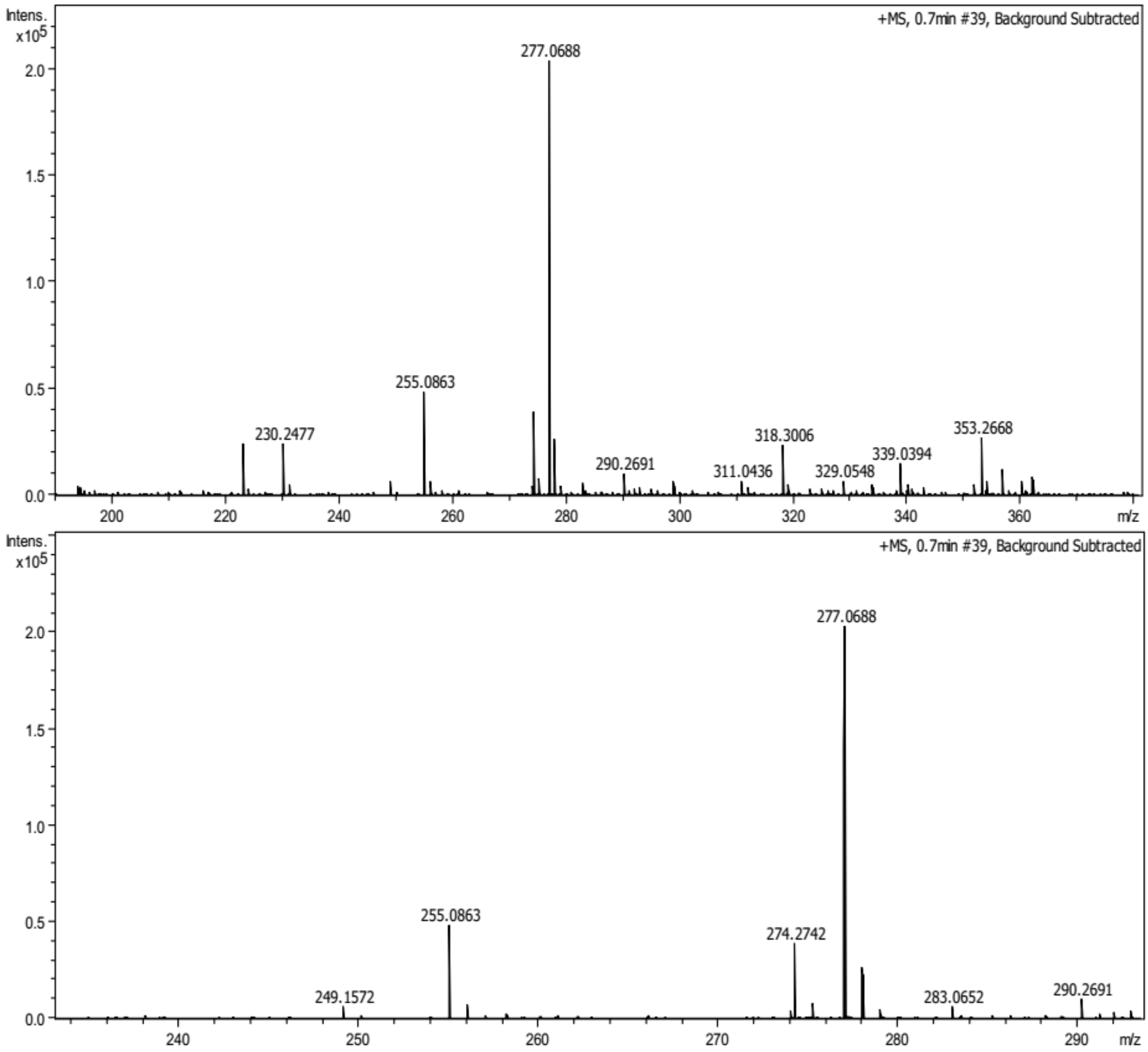

Meas. m/z	#	Ion Formula	m/z	err [ppm]	mSigma	# Sigma	Score	rdb	e⁻ Conf	N-Rule	
255.08631		C12H15O6	255.0863	-0.0	4.4	1	100.00	5.5	even	ok	($M+H^+$)
277.06881		C12H14NaO6	277.0683	-1.9	2.0	1	100.00	5.5	even	ok	($M+Na^+$)

Table S1. ^{1}H and ^{13}C NMR Data for compound **6** and 6,8-dihydroxy-7-methoxy-3-methyl-isocoumarin in CH_3OH-d_4

Position	**6**		**6,8-dihydroxy-7-methoxy-3-methylisocoumarin**	
	**δ_C, type**	**δ_H mult. (*J* in Hz)**	**δ_C, type**	**δ_H mult. (*J* in Hz)**
1	168.1, C	—	168.2, C	—
2	—	—	—	—
3	154.6, C	—	154.6, C	—
4	105.3, CH	6.29, s	105.3, CH	6.30, s
5	103.7, CH	6.37, s	104.0, CH	6.38, s
6	160.0, C	—	161.0, C	—
7	135.2, C	—	135.5, C	—
8	156.1, C	—	156.2, C	—
9	100.4, C	—	100.0, C	—
10	136.1, C	—	136.2, C	—
11	19.1, CH_3	2.22, s	19.1, CH_3	2.19, s
12	61.0, CH_3	3.86, s	60.9, CH_3	3.84, s

Table S2. ^{1}H and ^{13}C NMR Data for compound **7** and Diaporthein A in $CHCl_3$-d

Position	**7**		**Diaporthein A**	
	**δ_C, type**	**δ_H mult. (*J* in Hz)**	**δ_C, type**	**δ_H mult. (*J* in Hz)**
1	24.8, CH_2	1.70, m; 1.82, m	24.8, CH_2	1.70, m; 1.82, m
2	18.0, CH_2	1.61, m; 1.67, m	18.0, CH_2	1.61,m; 1.67, m
3	37.7, CH_2	1.22, m; 1.69, m	37.8, CH_2	1.22, m; 1.69, m
4	38.0, C	—	38.0, C	—
5	81.3, C	—	81.3, C	—
6	105.9, C	—	105.9, C	—
7	73.3, CH	4.63, d, (2.2)	73.3, CH	4.63, d, (2.1)
8	136.8, C	—	136.8, C	—
9	77.1,C	—	77.1, C	—
10	50.1, C	—	50.1, C	—
11	67.3, CH	3.86, dd, (12.1, 4.2)	67.3, CH	3.86, dd, (12.0, 4.2)
12	40.4, CH_2	1.71, m; 1.89, m,	40.4, CH_2	1.71, m; 1.89, m
13	38.4, C	—	38.3, C	—
14	133.3, CH	5.99, dd, (1.6, 1.6)	133.3, CH	5.99, dd, (1.6, 1.7)
15	146.4, CH	5.85, dd, (17.5, 10.6)	146.4, CH	5.85, dd, (17.5, 10.7)
16	111.5, CH_2	5.04, m	111.5, CH_2	5.04, m
17	25.3, CH_3	1.16, s	25.3, CH_3	1.16, s
18	29.6, CH_3	1.25, s	29.6, CH_3	1.26, s
19	24.3, CH_3	1.43, s	24.2, CH_3	1.44, s
20	68.4, CH_2	3.35, d, (9.7); 3.97, d, (9.7)	68.3, CH_2	3.36, d, (9.6); 3.97, d, (9.6)

Table S3. ^{1}H and ^{13}C NMR Data for compound **8** and Diaporthein B in $CHCl_3$-*d*

**Position**	**8**		**Diaporthein B**	
	**δ_C, type**	**δ_H mult. (*J* in Hz)**	**δ_C, type**	**δ_H mult. (*J* in Hz)**
1	25.2, CH_2	1.96, m; 2.03, m	25.2, CH_2	1.96, m; 2.03, m
2	17.7, CH_2	1.62, m; 1.68, m	17.6, CH_2	1.62, m; 1.68, m
3	37.5, CH_2	1.24, m; 1.56, m	37.5, CH_2	1.23, m; 1.55, m
4	37.3, C	—	37.3, C	—
5	81.9, C	—	81.9, C	—
6	104.1, C	—	104.1, C	—
7	196.3, C	—	196.2, C	—
8	134.6, C	—	134.7, C	—
9	76.2,C	—	76.2, C	—
10	51.1, C	—	51.1, C	—
11	67.7, CH	4.04, dd, (11.7, 4.1)	67.7, CH	4.03, dd, (11.7, 4.1)
12	39.9 , CH_2	1.73, m; 2.07, m,	39.9, CH_2	1.73, m; 2.07, m
13	40.0, C	—	40.1, C	—
14	150.5, CH	6.82, d, (1.9)	150.4, CH	6.81, d, (1.8)
15	144.0, CH	5.83, dd, (17.5, 10.5)	144.1, CH	5.82, dd, (17.5, 10.7)
16	113.1, CH_2	5.09, d, m	113.1, CH_2	5.09, m
17	25.9, CH_3	1.22, s	25.9, CH_3	1.22, s
18	27.0, CH_3	1.20, s	26.9, CH_3	1.19, s
19	23.6, CH_3	1.44, s	23.7, CH_3	1.45, s
20	68.6, CH_2	3.72, d, (10.2); 4.14, d, (10.1)	68.6, CH_2	3.71, d, (10.2); 4.14, d, (10.2)
